# A Novel Deep Learning Method for Intelligent Fault Diagnosis of Rotating Machinery Based on Improved CNN-SVM and Multichannel Data Fusion

**DOI:** 10.3390/s19071693

**Published:** 2019-04-09

**Authors:** Wenfeng Gong, Hui Chen, Zehui Zhang, Meiling Zhang, Ruihan Wang, Cong Guan, Qin Wang

**Affiliations:** 1 Key Laboratory of High-Performance Ship Technology of Ministry of Education in China, School of Energy and Power Engineering, Wuhan University of Technology, Wuhan 430063, China; wfgongcn@163.com (W.G.); zhangtianxia918@163.com (Z.Z.); rhan_wang@163.com (R.W.); guancong2008@gmail.com (C.G.); 2 Beihai Campus, Guilin University of Electronic and Technology, Beihai 536000, China; guetwqin@163.com

**Keywords:** intelligent fault diagnosis, convolutional neural network, support vector machine, global average pooling, multichannel, data fusion, deep learning, rotating machinery

## Abstract

Intelligent fault diagnosis methods based on deep learning becomes a research hotspot in the fault diagnosis field. Automatically and accurately identifying the incipient micro-fault of rotating machinery, especially for fault orientations and severity degree, is still a major challenge in the field of intelligent fault diagnosis. The traditional fault diagnosis methods rely on the manual feature extraction of engineers with prior knowledge. To effectively identify an incipient fault in rotating machinery, this paper proposes a novel method, namely improved the convolutional neural network-support vector machine (CNN-SVM) method. This method improves the traditional convolutional neural network (CNN) model structure by introducing the global average pooling technology and SVM. Firstly, the temporal and spatial multichannel raw data from multiple sensors is directly input into the improved CNN-Softmax model for the training of the CNN model. Secondly, the improved CNN are used for extracting representative features from the raw fault data. Finally, the extracted sparse representative feature vectors are input into SVM for fault classification. The proposed method is applied to the diagnosis multichannel vibration signal monitoring data of a rolling bearing. The results confirm that the proposed method is more effective than other existing intelligence diagnosis methods including SVM, K-nearest neighbor, back-propagation neural network, deep BP neural network, and traditional CNN.

## 1. Introduction

The health condition monitoring and fault diagnosis of rotating machinery are of great importance in modern industry [1,2]. Rotating machinery is one of the most important types of engineering equipment [3]. In the past decade, with the rapid development of high-performance rotating machinery, such as advanced supersonic vector aircraft engines, large generator sets, precision machine tool spindles, and high-performance marine propulsion motor, etc., are developing increasingly toward automation, unmanned, and ultrahigh speeds [4]. To ensure the safety and reliability of rotating machines’ operation, it is necessary to establish a highly efficient and intelligent fault diagnosis and health monitoring system [5]. In general, significant faults are gradually evolved from incipient micro-faults. Incipient faults have a smaller effect on the stability of rotating machinery, and it is easy to handle [6]. However, the features of incipient faults are not obvious, and the detection and diagnosis of micro-faults are more difficult than the significant faults. Nowadays, incipient micro-fault diagnosis and monitoring methods are widely investigated by scholars in the field of fault diagnosis [1,6,7,8,9]. 

In general, fault diagnosis methods may be divided into two types: mechanism analytical model methods [10] and data-driven methods [11]. The former needs to establish a high-precision mathematical model to describe the evolution mechanism of a fault. Although this method can obtain better results, it is difficult to establish a high-precision complex system model. In addition, the established model is difficult to transplant to solve other similar problems [6]. Therefore, with the increasing complexity of the mechanical system, the fault diagnosis methods based on mechanism analytical models are restricted in actual applications. Nowadays, with the rapid rise and popularity of the “internet+” and the internet of things, the advanced intelligent sensors and data acquisition technology are widely used in rotating machinery and equipment [4,5]. The massive monitor data such as the vibration, noise, temperature, current, and pressure of rotating machinery are easily obtained, and those historical data records the life cycle health information of rotating machinery from the beginning to end of service [5]. Therefore, engineers may perform a fault diagnosis by the statistical analysis of huge historical data. In recent years, the data-driven fault diagnosis method is widely popularized and applied [11]. For example, the Yangtze Three Gorges Hydropower Station of China has about 60 large hydroelectric generator sets. Each generator set needs to monitor multiple condition indicators including vibration, noise, pressure pulsation, efficiency, bearing temperature, generator temperature, generator air gap, generator partial discharge, transformer oil and gas, etc. [12]. The monitoring data of each indicator is up to terabytes (TB). Therefore, it is unrealistic to rely on the experience of engineers or experts only to analyze faults by extracting features manually. This situation is very common in many other fields, such as aircraft engines, smart ships, unmanned vehicles, and autonomous ships. Thus, it is extremely necessary to establish intelligent, automatic, and adaptive data-driven fault diagnosis methods in the context of big data. 

In recent years, with the gradual rise of machine learning and deep learning research, fault diagnosis methods based on artificial intelligence (AI) have gradually become a hot topic [1,2,3,4,5]. Different from the traditional fault diagnosis methods based on signal processing technology, the intelligent diagnosis algorithm can automatically extract the useful representative features from monitoring data [2]. The traditional intelligent diagnosis method usually includes three steps: feature extraction, feature selection, and fault classification [3]. Feature extraction is to process and transform the raw data signals collected by the multiple sensors in the time domain, frequency domain, and time-frequency domain and to extract useful representative features for subsequent fault identification [13,14,15,16]. The commonly used feature extraction methods include wavelet transform (WT) [13], spectral analysis (SA) [14], empirical mode decomposition (EMD) [15], and Fourier transform (FFT) [16]. Feature selection further filtrates and removes the low sensitivity and useless data from the extracted features. The common feature selection methods mainly include principal component analysis (PCA) [8] and independent component analysis (ICA) [9]. Fault classification puts the selected features input into the fault classifier for pattern recognition and, finally, outputs the classification results through repeated iterative training [1,2,3]. Back-propagation artificial neural network (BPNN) [17], support vector machine (SVM) [18,19], and K-nearest neighbor (KNN) [20] have become the most popular fault classifier in the past decade [1]. Through application and verification, the above three algorithms have a poor feature extraction ability due to their shallow network structure, and it is difficult to be applied to other devices in the context of the big data [5]. At present, some scholars usually combine manual feature extraction with shallow machine learning algorithms to perform an intelligent fault diagnosis [17,18,19,20].

The abovementioned existing intelligent fault diagnosis methods have been applied and obtained certain effective results; however, there still exist four major shortcomings: (1) The massive monitoring signals collected from rotating machinery under different working conditions are always very complex and non-stable with heavy background noises [3]. (2) Engineers must master various advanced signal processing techniques for data preprocess and feature extraction. However, the feature selection excessively relies on the experience and prior knowledge of engineers [4]. Furthermore, it is very subjective, time-consuming, and blind [3]. (3) The extracted features are mainly used to solve specific fault problems, and the versatility is poor [21]. Different fault types, fault locations, and fault severities will affect the effect of feature extraction [3,21]. Also, manual feature extraction and selection methods are difficult to adapt to the millions of samples of big data. (4) Manual operation is difficult to extract the micro-features of an incipient fault. As the feature of an incipient fault is not very distinct and would be covered by background noise easily, engineers may try hard to detect the micro-fault and may delete it as noise. The inherent shortcomings mentioned above are derived from the shallow model architectures employed in the traditional intelligent diagnosis methods. It would lead to an insufficient feature extraction ability [3]. Consequently, there is an urgent need to develop deep network model structures used for automatic data deep mining, feature extraction, and intelligent fault diagnosis of rotating machinery.

In 2006, Hinton and Salakhutdinov jointly proposed the deep learning theory [22]. Deep learning is a great breakthrough in the artificial intelligence field [3]. It uses the deep neural network architectures to extract representative features layer by layer from the input data and automatically and greedily learns the inner relation between the input data and labels [23]. One of the most important advantages of deep learning methods is that they can automatically learn the representative features and the complex nonlinear relationships from the raw data [24]. In addition, it can greatly get rid of the long-term dependence of manual feature extraction and selection based on expert experience [3]. Currently deep learning technology has been successfully applied in the fields of speech recognition [25], image recognition [26], and natural language processing [27]. The recognition ability of images and the speech of the deep learning model is over that of human beings. The powerful feature extraction capability of deep learning has the potential to identify micro-fault features and to overcome the aforementioned inherent defects of traditional intelligent diagnostic methods [1,2,3,4].

Since 2013, deep learning method and deep neural networks (DNN) have attracted more and more attention and have been gradually applied in the mechanical fault diagnosis field. Jia et al. [28] proposed a five-layer DNN model (including three hidden layer) by using the deep auto-encoder method for an intelligent fault diagnosis of rolling bearings and planetary gearboxes, but the raw data needed to be transformed into frequency spectra. Tamilselvan et al. [29] proposed a DNN method based on a deep belief network used for an intelligence fault diagnosis of aircraft engines and power transformers. Li et al. [30] proposed a 3-layer DNN based on the deep belief network (DBN) for a fault diagnosis of rolling bearings. However, in their bearing experiments, the fault was simulated by grooving, so the fault features obviously were a significant fault, which showed difficulty in reflecting the effectiveness and ability of their model when used for dealing with a micro-fault diagnosis. 

Convolutional neural network (CNN) or CNNs were first proposed by LeCun et al. [31]. It is one of the most important branches of deep learning [2]. Compared with the other DNN, CNN model has fewer parameters due to shared filters [23]. CNN has a powerful feature extraction ability and is mainly used in image recognition [26]. In recent years, some scholars have applied CNN in the field of fault diagnosis. Xia et al. [4] proposed a DNN method by stacking two groups CNN model used for intelligence fault diagnosis of bearings and gearboxes. However, their method still needed the frequency spectra of raw data. Verstraete et al. [32] proposed a deep learning method by using the CNN method for a fault diagnosis of rolling element bearings. Similarly, their methods still needed to turn raw data into a time-frequency image. Although the above studies all used CNN algorithm, they still needed the traditional feature extraction method to extract the features from raw vibration data. It has not made full use of the CNN’s powerful feature extraction ability and has limited the further improvement of the effectiveness of a fault diagnosis. Recently, Zhang et al. [33] directly put a 2-D representation of raw vibration signals input into a CNN model for the fault diagnosis of bearings. Although their methods were without the manual feature extraction from raw data, a shortcoming still existed in their research. In their CNN model, the fully connected network structure was used. As the parameter quantity of the fully connected structure in CNN model was too large, it led to a long time duration for training and testing [2,4]. This shortcoming would have a negative impact on a rapid fault diagnosis and the real-time detection of micro-faults. 

For the aforementioned shortcomings, in this paper, a novel method called the improved global average pooling CNN-SVM algorithm is developed for the intelligent diagnosis of incipient faults in rotating machinery. The proposed method improves the traditional CNN model structure, using a combination of a 1 × 1 convolution layer and a global average pooling layer [34] to replace the fully connected layer of 2–3 layers and using the SVM to replace the Softmax function in test stage. The deep learning training skills are used to improve the algorithm generalization ability and to prevent model over-fitting. The proposed method is divided into three main steps: Firstly, the temporal and spatial multichannel raw fault data from multiple sensors are directly input into the improved CNNs-Softmax model to complete the training of the CNN model. Secondly, the improved CNNs are used as feature extractors to extract representative features from the new fault raw data. Finally, the extracted sparse representative feature vectors are input into the SVM used for fault classification. The proposed CNN-SVM method does not use any manual feature extraction and signal processing on the raw vibration data. It can get rid of the dependence on expert experience and prior knowledge. In this paper, the proposed method is applied to the diagnosis of rolling bearing 2-channel experimental vibration signal data and is compared with traditional intelligent diagnosis methods including SVM, KNN, BPNN, deep BP neural network (DBPN), and traditional CNN. The results confirm that the proposed method is more effective than the other existing intelligent methods. 

There are two main contributions in this paper. Firstly, a novel method of intelligent fault diagnosis based on the combination of CNNs as a feature extractor and SVM as a fault classifier is proposed. Multichannel 2-D raw input data based on multiple sensors are used as the input of CNN-SVM. At the same time, the category and quantity of misclassification is visualized and quantified in the proposed model by using a multi-class confusion matrix. Secondly, by improving the traditional CNN network structure, the global average pooling layer is designed to replace the structure of the fully connected network in CNN, which reduces the training parameter amount and calculation time. Furthermore, the robustness and generalization of the proposed algorithm is improved by using various deep learning training techniques such as data enhancement, dropout, and batch normalization. The proposed method is more suitable for online monitoring and real-time rapid diagnosis of faults. 

The rest of this paper is organized as follows. The basic theory and structure of CNN is briefly introduced in Section 2. In Section 3, the intelligent diagnosis method based on an improved CNN-SVM is described in detail. In Section 4, the experimental results are analyzed and discussed. Finally, the conclusions are given in Section 5. 

## 2. Standard Convolutional Neural Network

**Figure 1:**
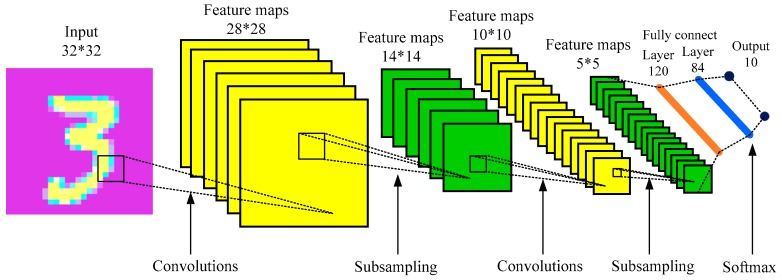
The basic structure of the convolutional neural network [31].

A convolutional neural network is a deep feed-forward neural network model that is used to process data with mesh-like structures [23]. The CNN was first proposed by Yann LeCun in 1989 and is mainly used for handwritten figure recognition [31]. CNN has powerful feature extraction abilities by constructing multiple filters and uses these filters to extract the representative features from input data layer by layer [2]. It combines the sparse connection with the parameter weight sharing mechanism, and the data dimension is down-sampled in time and space, which greatly reduces the amount of training parameters and effectively avoids an over-fitting of the algorithm [35]. In CNN model, the back-propagation (BP) algorithm is used to update the model parameters [24,35,36]. Due to its good adaptability to the scaling, tilting, and translation of images, it is great widely applied in image recognition and other similar problems [24,26,32,33,36]. A basic structure of CNN is shown in Figure 1 [31].

CNN is a multistage stacked neural network [4]. The simplest CNN model is composed by one trainable feature extraction stage and one classification stage [2]. Each stage involves linear and nonlinear operations, and each stage has a different role. The feature extraction stage contains three kinds of layers: the convolutional layer, the activation layer, and the pooling layer [23]. A deep CNN network can be constructed by alternately stacking multiple feature extraction stages. The feature extraction stages are used for extracting representative features from the input data layer by layer. The classification stage is a multilayer perceptron consisting of several fully connected layers [33]. The input and output of each stage are sets of matrices called feature maps [37]. The feedforward calculation process [4] can be written as:

(1) $$f(X)=f_{N}(\cdots f_{3}(f_{2}(f_{1}(X,\theta^{\left(1\right)}),\theta^{\left(2\right)}),\theta^{\left(3\right)}),\cdots ),\theta^{\left(N\right)})$$

where $X=\left[x_{1},x_{2},\cdots ,x_{m}\right]\in R^{1\times m}$ is the input feature map of raw input data, such as 2-D image data or 1-D time series data including vibration, audio, pressure, and current signals from a mechanical health monitoring system [5]; $\theta^{\left(1\right)},\theta^{\left(2\right)},\cdots ,\theta^{\left(N\right)}$ are the training parameters including weights and biases; $f_{1},f_{2},\cdots ,f_{N}$ are the linear or nonlinear operations at each stage; and f(X) is the final output of the original data X obtained by several convolution, activation, and pooling operations, etc. 

### 2.1. Convolution Operation Layer 

**Figure 2:**
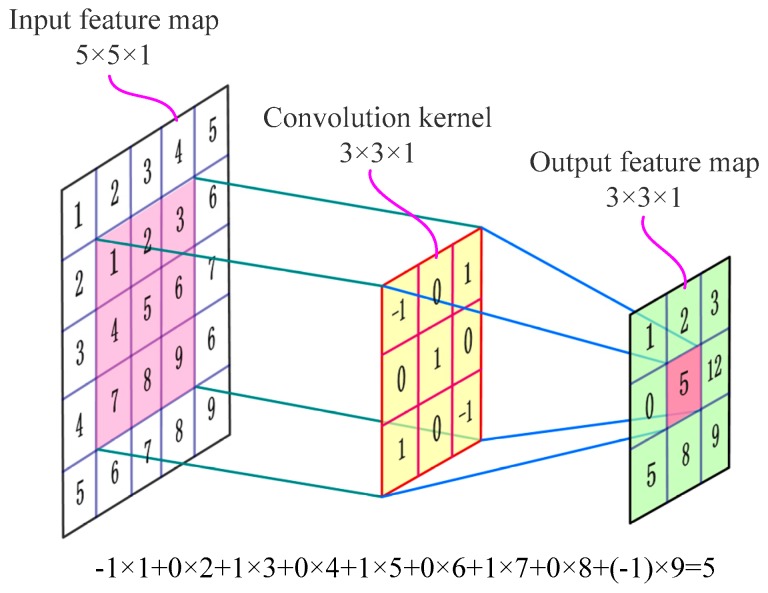
A schematic diagram of a convolution operation.

The convolutional layer is one of the most important core modules in CNN [2]. It contains multiple convolution kernels (also known as filters). The filter is usually a rectangular matrix, and each value of the matrix is a weight (also known as a characteristic value) learned from the input data [23]. Each filter has different weights. These weights are calculated automatically by an error back propagation algorithm [4]. In the convolutional layer, the input feature map is convolved with a bunch of learnable convolution kernels to generate many new feature maps as the input to the next layer [4]. The calculation method principle schematic diagram of a convolution operation is shown in Figure 2. In Figure 2, the left picture is an input feature map, the middle is a convolution kernel, and the right is an output feature map. 

In Figure 2, the convolution kernel can be moved on the input feature map along with the up-down and left-right directions according to the stride [23]. Usually, the stride is larger than or equal to 1. Multiply the corresponding element values of the coincident region between the convolution kernel and input feature map and then add the offset to obtain a value of output feature map. In the feedforward calculation process, the output feature maps of the convolutional layer can be obtained by using the rectangular convolutional kernel to traverse each element on the entire input feature map. The convolution operation of a multichannel input feature map can be described as follows [4]:

(2) $$X_{i}^{\left(k\right)}=\sum_{c=1}^{C}W_{i}^{\left(c,k\right)}⊗X_{i-1}^{\left(c\right)}+B_{i}^{\left(k\right)}$$

where ⊗ represents the convolutional operator; *i* denotes the index of the network layer; *k* represents the index number of the convolution layer output feature maps and also marks the *k*th group convolution kernel; each group contains C convolution kernel; *k* = 1, 2, …, K; *c* = 1, 2,…, C and is the channel index number of input feature maps; $X_{i-1}^{\left(c\right)}$ is the input feature map of channel c; $X_{i}^{\left(k\right)}$ is the *k*th output feature map after the convolution calculation of the *k*th group convolution kernel and the input feature map; $W_{i}^{\left(c,k\right)}$ is the weight of the convolution kernel at the *i*th layer, the filter of *c*th channel in the *k*th group convolution kernel; and $B_{i}^{\left(k\right)}$ is the bias of the *k*th group filter in the *i*th layer. If C = 3, it represents the input feature map, a 3-channel array such as a color picture with three Red-Green-Blue channels. 

### 2.2. Activation Operation Layer

After the convolution operation, a nonlinear transformation activation function is applied to the output of the convolution layer [2]. The purpose of the activation layer is to improve the expression ability of the model. Different activation functions can obtain different nonlinear transformations. Equation (2) can be rewritten as follows after adding the activation function:

(3) $$A_{i}^{\left(k\right)}=f\left(X_{i}^{\left(k\right)}\right)=f\left(\sum_{c=1}^{C}W_{i}^{\left(c,k\right)}⊗X_{i-1}^{\left(c\right)}+B_{i}^{\left(k\right)}\right)$$

where f(⋅) is the activation function and $A_{i}^{\left(k\right)}$ is the *k*th output feature map after a nonlinear transformation by the activation layer. 

In CNN, the most commonly used activation functions include the Sigmoid, hyperbolic tangent, and rectified linear unit (ReLU). Their expressions and graphs are shown in Table 1. More activation functions can be seen in Reference [3]. As can be seen from Table 1, the sigmoid function is similar to the tanh function. The input value *x* of both sigmoid and tanh function is −∞~+∞, and the output value f(x) of both the sigmoid and tanh functions respectively are 0–1 and −1–1. Both of these functions have better nonlinear transformation capabilities, but the inherent disadvantage is a gradient disappearing and gradient saturation problem [38]. When the absolute value of the input value *x* is large, the output change values f(x) of the two functions almost is zero. In order to solve this problem, the ReLU activation function only considers the forward signal, ignoring the influence of the negative signal, and it has a good fitting ability and sparsity, which greatly improves the efficiency of the calculation [2]. The ReLU function can effectively prevent a gradient disappearance and over-fitting and has been widely used in recent years [35].

### 2.3. Pooling Operation Layer 

**Figure 3:**
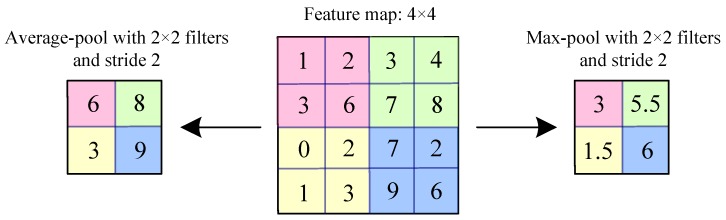
A schematic diagram of a pooling operation.

The pooling layer (also known as subsampling or down-sampling) is located behind the convolutional layer. The main role of pooling is to reduce the dimension and quantity of trainable parameters of a CNN and to screen the main representative features from the activation layer output feature maps [2,4,24]. Usually, the convolution layer mainly relies on the increasing number of output channels to improve the feature extraction ability of the model. Each output channel corresponds to a filter. Each filter can only extract one type of feature because of sharing parameters [23]. Therefore, as many potential features as possible are extracted from the raw data using many filters in a CNN. As the convolution operation increases the number of output feature maps, the feature dimension will be increased sharply, so the dimensionality disaster is easily caused [39]. The pooling operation mainly uses a rectangular pooling kernel (also known as a pooling window) for feature screening [24]. The operation of pooling is similar to convolution. The pooling window can be set to different sizes, and the pooling kernel can also be moved on the feature map along with the up-down and left-right directions according to the stride [23]. The stride is larger than or equal to 1. The calculation method principle schematic diagram of pooling operation is shown in Figure 3.

Different from the convolution operation, in Figure 3, the pooling operation generates a representative value to replace all elements in the pooling window. Pooling operations mainly include maximum pooling and average pooling [23,33]. In Figure 3, the middle graph is the raw feature map, the left is the average pooling operation, and the right is the maximum pooling operation. The maximum pooling (max-pooling) selects a maximum value as the representative value from all the elements in the corresponding area of the pooling window [35]. Average pooling calculates the average value from all the elements as the representative value in the corresponding area of the pooling window. Max-pooling is excellent at handling texture features, and average pooling is more sensitive to background information [23]. In CNN, the max-pooling is widely used in a pooling layer [35]. The mathematical expression of max-pooling [33] is described as follows:

(4) $$P_{i}^{\left(k\right)}=\underset{\begin{array}{l} (j-1)S+1<t_{x}\leq jS\\ (j-1)H+1<t_{y}\leq jH \end{array}}{\max}\left\{A_{i}^{\left(k\right)}\left(t_{x,y}\right)\right\}$$

where $A_{i}^{\left(k\right)}\left(t_{x,y}\right)$ represents the $t_{x,y}$ th pixel value in the *k*th output feature map of the *i*th layer, *S* and *H* are the width and height of the pooling window respectively, $\left\{A_{i}^{\left(k\right)}\left(t_{x,y}\right)\right\}$ is a matrix of the shape *S* × *H*, and $P_{i}^{\left(k\right)}$ is the output feature map after the pooling operation.

### 2.4. Fully Connection Layer 

Fully connection layer is used in the classification stage in traditional CNN architecture. The main role is to further extract the features of the output data of CNN and to connect the feature extraction stages with the Softmax classifier [4,34]. In the traditional CNN, a fully connection layer is usually composed of 2~3 layers fully connected to a feedforward neural network. The final output feature map of a CNN is transformed into a one-dimensional array by a Flatten function. This one-dimensional array is thought as the input of the fully connection layer. Finally, the output of the fully connection layer is a one-dimensional vector. Each value of 1-D vector is a quantitative value of n classifications. In a fully connected network, all neurons between layers are interconnected, with the following definition:

(5) o(X)=f(W⋅X+B)

where f(⋅) denotes the activation function, *X* is the input of a fully connected layer, o(X) is the output of a fully connected layer, and *W* and *B* are the weights and biases of a fully connected network, respectively. 

In the conventional CNN network shown in Figure 1, the classification output layer is usually composed of a fully connected layer and a Softmax classifier [4,33,39]. However, the inherent shortcoming of a fully connected layer has too many trainable parameters [4]. In the traditional CNN, the parameter quantity of the fully connected layer accounts for 80–90% of the total parameter quantity of the CNN model [34,40]. This shortcoming offsets the advantages of pooling operations to reduce the dimensionality and parameters in a large extent. Moreover, the number of parameters increases exponentially with the increasing number of fully connected layers. Therefore, the structure of the fully connected layer in a CNN not only occupies too many computing resources but also easily leads to model over-fitting [34]. Worse, the test time will be too long when using the trained fully connected CNN model to online diagnose the faults, leading to it being not suitable for applications with rapid diagnosis and real-time online detection. Another disadvantage of the traditional CNN network is that the Softmax function [41] is used as the final classifier. The Softmax classifier essentially transforms the final classification result into a normalized operation that conforms to the probability distribution [4]. It is not as powerful as SVM in a multi-class classification performance. The SVM has powerful multi-class classification capabilities in reality [42]. However, the shortcoming of SVM is that its capabilities of deep feature extraction and data mining are insufficient, and SVM is more suitable for processing small sample data [1,6]. Therefore, it is difficult to play a more superior performance in the diagnosis of micro-faults and big data samples. 

## 3. The Improved CNN-SVM Intelligent Fault Diagnosis Method

### 3.1. Improved CNN-SVM Algorithm Construction 

**Figure 4:**
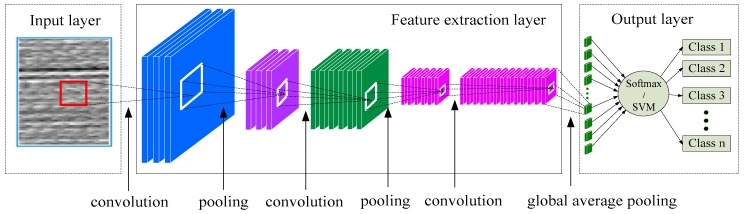
The model structure of the improved CNN-support vector machine (SVM) method.

By analyzing the shortcomings of a traditional CNN algorithm construction, this paper improves the traditional CNN model structure. Firstly, the global average pooling technology is introduced to replace the fully connected network structure in CNN, which effectively reduces the parameter quantity and calculation time of the CNN model. Secondly, the nonlinear SVM is used to replace the Softmax function in the test stage, which effectively improves the accuracy of the classification results. The model structure of the improved CNN-SVM method is shown in Figure 4. 

**Figure 5:**
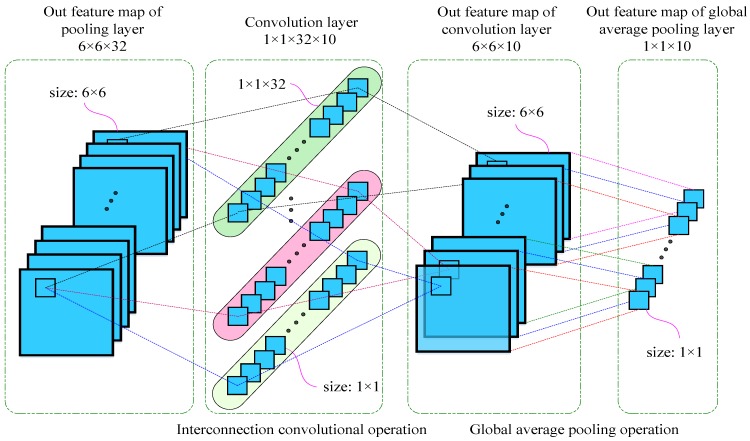
The structure design of the improved CNN model.

In the improved CNN-SVM algorithm, it is mainly composed of an input layer, a feature extraction layer, and a classification estimation output layer. The feature extraction layer is the core part of the CNN-SVM algorithm. It consists of multiple convolutional layers, activation layers, and pooling layers stacked in sequence. Different from a traditional CNN, the proposed method uses a combination of a 1 × 1 convolution layer [43] and a global average pooling layer [34] to replace the 2~3 layers of fully connected layers. A detailed structural design is shown in Figure 5. 

In Figure 5, it is assumed that there are 10 classification categories. In a traditional CNN, the output of the fully connection network should be 1 × 10. Here, it is assumed that the given size of an output feature map of the bottom pooling layer is 6 × 6 × 32, where 6 × 6 denotes the size of an image pixel is 6 × 6 and where 32 represents the channel number of a pooling layer output feature map. In the traditional CNN model, the 6 × 6 × 32 multidimensional array is usually converted into a one-dimensional array, which is considered as the input of the fully connection layer by a Flatten function. The hidden layer of a fully connection layer is usually 2–3 layers [4]. The number of nodes in the last hidden layer is set to be the number of categories of the classification. In this case, the final output dimension is 10. Assuming that the number of nodes of the 2 hidden layers are 512 and 256, respectively, the total amount of learnable parameters of fully connection layer is up to 724,234. The detailed calculation process is (6 × 6 × 32 + 1) × 512 + (512 + 1) × 256 + (256 + 1) × 10 = 724,234. This is just a small-scale example, and the total amount of learnable parameters in the actual engineering is much larger. Therefore, the full connection part occupies a huge computing resource. The calculation effective of a traditional CNN is inefficient due to defects in the fully connected layer when processing large-size images [34]. To solve this problem, the proposed method replaces the fully connected part with global average pooling technology. The output format of a fully connection layer is 1 × 10, so the output feature map size of the global average pooling should also be 1 × 10. To facilitate a CNN calculation, the format of 1 × 10 is transformed into a 2-D image format of 1 × 1 × 10. The output feature map shape of the global average pooling layer is 1 × 1 × 10, and the shape of the input feature map is 6 × 6 × 32. The goal is to convert 6 × 6 × 32 into 1 × 1 × 10 and to finally get a 1 × 10 output, as shown in Figure 5. The detailed improvement steps are described as follows:

Firstly, the shape of 6 × 6 × 32 is converted into 6 × 6 × 10. In order to accomplish this task, a transitional convolution layer of 1 × 1 × 32 × 10 is specially designed. The size of a convolution kernel is 1 × 1, the stride is 1, and the padding mode is “Same”. The role of the transitional convolutional layer is to change only the number of output channels of the input feature map without changing its image size. The 6 × 6 × 32 feature map is calculated by 1 × 1 × 32 × 12 convolution layers, and a feature map of 6 × 6 × 10 is obtained. In Figure 5, the input channel is 32, and the output channel after convolution calculation is 10. Therefore, if it wants to reduce the number of channels of the 32-channel feature map to 10, it must set up 10 groups of convolution kernels. Also, the number of each group convolution kernels should be 32 because the channel number of the input feature map is 32. Therefore, in each group convolution kernel, there are 32 convolution kernels of 1 × 1. The 32 1 × 1 filters are calculated by a convolution with 32 6 × 6 input feature maps, one each. The 32 6 × 6 feature maps are still obtained because the 1 × 1 convolution operation does not change the input feature map size. Then, the corresponding elements of the 32 6 × 6 feature maps are summed to obtain a total 6 × 6 feature map. Ten groups of 1 × 1 × 32 convolution kernels correspond to the 10 output feature maps. Finally, a 6 × 6 × 10 output feature map is obtained, as shown in Figure 5. 

Secondly, the shape of 6 × 6 × 10 is converted into 1 × 1 × 10. In order to realize this goal, a global average pooling technology is applied. The 10 feature maps of 6 × 6 correspond to 10 global average pooling kernels. Each pooling kernel size of the global average pooling layer is 6 × 6, the stride is 6, and the padding mode is “Same”. The role of the global average pooling layer is to change only the size of feature map without changing its number of output channel [34]. The 36-pixel values of each 6 × 6 feature map are averaged to obtain a global average value. According to this method, 10 feature maps of 6 × 6 can obtain 10 output values of 1 × 1. Therefore, the final output feature map is 1 × 1 × 10, as shown in Figure 5.

The improved CNN model using the global average pooling method is better than the traditional CNN model used the fully connected layer [34]. The reason is that, on the one hand, the global average pooling technique greatly reduces the number of training parameters of the CNN model and reduces the risk of CNN model over-fitting. In this case, the total parameter amount of global average pooling part is only 330. The calculation process can refer to Equation (19). However, the total parameter amount of fully connection layer is up to 724,234. Also, the global average pooling has no trainable parameters. On the other hand, the improved CNN model reduces the number of network layers of the original CNN model and avoids the gradient disappearance problem caused by the fully connected multilayer perceptron. Therefore, the train efficiency and calculation speed of the improved CNN model is more efficient than the original CNN model. Behind the improved CNN model, the Softmax layer and the SVM classifier are designed to be connected in parallel, are located in the improved CNN model, and finally construct the CNN-SVM model. The basic components of the CNN-SVM algorithm are as follows: 

(1) Raw data input layer 

**Figure 6:**
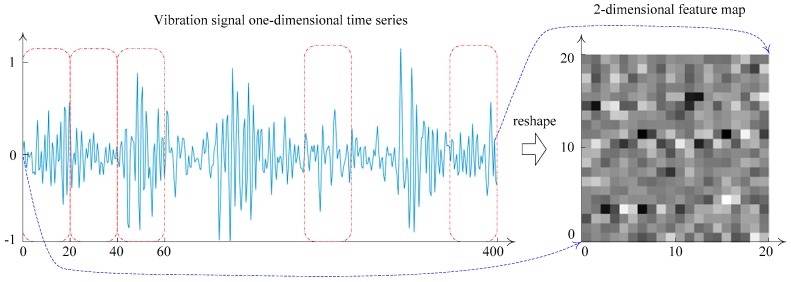
The procedure of data reconstruction.

The input layer is used for receiving the raw fault monitoring data from the rotating machinery, and some necessary operations are conducted on the raw data such as data standardization and format normalization. The raw data need to be converted to the trainable type of CNN [4]. In general, the input data format of a CNN model is 2-D pixel grid data (such as image data) or 3-D data (such as a CT scan or color video data) [23,35]. In this paper, the one-dimensional time series fault vibration signal data of the rotating machinery can be transformed into a two-dimensional input feature map form by the data reconstruction method [44]. The data reshaping process is shown in Figure 6. 

(2) Feature extraction layer 

The feature extraction layer is the same as in the traditional CNN model. It consists of the convolutional layer, active layer, and pooling layer. A deep feature extraction layer could be constructed by alternately stacking multiple convolution layers, activation layers, and pooling layers according to the actual needs of the diagnostic object. A detailed description of the convolutional layer, activation layer, and pooling layer are found in Section 2.1, Section 2.2 and Section 2.3. 

(3) Global average pooling layer

The global average pooling layer is a new technique to solve the problem of too many learnable parameters of a fully connected network in a traditional CNN [34]. Similar to the pooling layer operation, global average pooling calculates a global average value from the whole output feature map of the last convolution layer. The size of a pooling kernel is the same as the size of a feature map. The value of the stride is the same as the size of pooling kernel, and the padding mode is “Same”. The detailed contents of the global average pooling technique have been introduced above. For the n-class classification problem, the number of output channels of the last convolutional layer is n, and n output feature maps are obtained. Through the global average pooling operation, each output feature map gets a representative value. Finally, the output of the global average pooling layer is a matrix of 1 × n. The mathematical expression of the global average pooling can be rewritten as:

(6) $$S_{avg-pooling}^{l}=\frac{1}{c}\sum_{i=1}^{c}X_{1:h,\;1:w,\;i}^{l}$$

where $S_{avg-pooling}^{l}$ represents the calculated result by the global average pooling of the *l*th feature map; *l* is the index of feature maps; *c* represents the total number of element values in the global average pooling kernel; 1:*h* denotes that the range of a pooling kernel in the height direction is from the 1st line to the *h*th line; 1:*w* indicates that the range of a pooling kernel in the width direction is from 1st column to the *w*th column; similarly, the *h* and *w* respectively represent the height and width of an output feature map of the last convolution layer; and $X_{1:h,1:w,\;i}^{l}$ represents the element value corresponding to the global average pooling kernel. 

(4) Softmax function layer

The Softmax function is an extension of the Logistic regression and mainly solves the multi-class classification problem [4,41]. The output result of a fully connection layer or the global average pooling layer is a quantization matrix ${\left\{Y\right\}}_{m\times n}$ of m rows and n columns. The *m* represents m samples, and the *n* represents the n quantized value corresponding to n categories. ${\left\{Y\right\}}_{m\times n}={\left\{Y_{1},\cdots Y_{i},\cdots ,Y_{m}\right\}}^{Τ}$, $Y_{i}=(y_{i}^{\left(1\right)},\cdots y_{i}^{\left(j\right)},\cdots ,y_{i}^{\left(n\right)})$, where $y_{i}^{\left(1\right)}$ indicates that the *i*th sample belongs to the probability value of the first class. The different element value in ${\left\{Y\right\}}_{m\times n}$ has different magnitudes which do not conform to the probability distribution [2]. In order to solve this problem, the Softmax function is usually used to normalize the calculation. After a Softmax normalization, the output value conforms to the probability distribution [4]. Assuming that the training input sample is *x* and the corresponding label is $\bar{y}$, the sample x is predicted to be the probability of category *j*, which can be defined as P(y=j | x). Here, n categories can obtain n quantized probability values. Each quantized value has different magnitudes. Then, the n probability values are input into the Softmax function. Finally, the output of Softmax layer is the n-dimensional vector that conforms to the probability distribution. The Softmax function mathematical expression is defined as [4,39]

(7) $$Y_{i}^{\;\prime }={\left[\begin{array}{c} y_{i}^{(1)\;\prime }\\ y_{i}^{(2)\;\prime }\\ \vdots \\ y_{i}^{(n)\;\prime } \end{array}\right]}^{Τ}={\left[\begin{array}{c} P(y_{i}=1\;|\;x_{i})\\ \begin{array}{c} P(y_{i}=2\;|\;x_{i})\\ \vdots \end{array}\\ P(y_{i}=n\;|\;x_{i}) \end{array}\right]}^{Τ}=soft\max(Y_{i})=\frac{1}{\sum_{l=1}^{n}e^{x_{{}_{i}}^{Τ}\cdot w_{l}}}{\left[\begin{array}{c} e^{x_{{}_{i}}^{Τ}\cdot w{\;}_{1}}\\ \begin{array}{c} e^{x_{{}_{i}}^{Τ}\cdot w_{\;2}}\\ \vdots \end{array}\\ e^{x_{{}_{i}}^{Τ}\cdot w{\;}_{n}} \end{array}\right]}^{Τ}$$

where $Y_{i}^{\;\prime }$ is the output value of the *i*th sample after normalization by Softmax; $Y_{i}^{\;\prime }=(y_{i}^{(1)\prime },\cdots ,y_{i}^{(l)\prime },\cdots ,y_{i}^{(n)\prime })$; the range of each value of $Y_{i}^{\;\prime }$ is from 0 to 1 and $\sum_{l=1}^{n}y_{i}^{(l)\prime }=1$, conforming to the probability distribution; $P(y_{i}=1\;|\;x_{i})$ is the probability value of the *i*th sample, belonging to category 1; $e^{x_{{}_{i}}^{Τ}\cdot w{\;}_{1}}$ represents converting $y_{i}^{\left(1\right)}$ to a value between 0 and 1; $1/\sum_{l=1}^{n}e^{x_{{}_{i}}^{Τ}\cdot w_{l}}$ is a normalization function; and the maximum value of each row in $Y_{i}^{\;\prime }$ is the fault category predicted of the CNN model. 

The loss error can be calculated by comparing the normalized prediction result with the corresponding sample actual label. The commonly used loss function includes the mean square error (MSE) loss function and the cross-entropy (CE) loss function [4]. In this paper, the cross-entropy loss function is applied because the input labels belong to the classification flag [39]. The cross-entropy cost function is defined as

(8) $$J(w)=-\frac{1}{m}[\sum_{i=1}^{m}\sum_{j=1}^{n}I\left\{{\bar{y}}_{i}=j\right\}\log\frac{e^{x_{i}^{Τ}\cdot w_{j}\;}}{\sum_{l=1}^{n}e^{x_{i}^{Τ}\cdot w_{l}}}]$$

where *i* represents the *i*th training sample, *j* represents the *j*th category (the total number of categories is n), and I{⋅} is a logical indication function. If the value in the brackets is true, I = 1, else I = 0. ${\bar{y}}_{\left(i\right)}$ is the actual label of the *i*th sample. $e^{x_{i}^{Τ}\cdot w_{j}\;}/\sum_{l=1}^{n}e^{x_{i}^{Τ}\cdot w_{l}}$ is a probability value calculation function by Softmax normalized, representing the probability value of the ith sample belonging to the *j*th category. J(w) is a cross-entropy loss function. The training process of CNN is to constantly adjust the parameters in Equation (8) to minimize the cost function J(w). 

(7) SVM classifier 

SVM is a classic two-class model [19]. It exhibits many unique advantages in solving small sample, multi-class, nonlinear, and high-dimensional pattern recognitions [1,42]. In this paper, the proposed CNN-SVM method mainly uses a nonlinear SVM as the backend multi-class classifiers. The nonlinear SVM is applied to solve the nonlinear multi-classification tasks. Through transforming linear indivisible problems into linear divisible problems by using nuclear skills and soft interval maximization techniques, the linear SVM classification algorithm is used to solve the nonlinear classification problem [42]. Usually, the fault data of rotating machinery are linearly inseparable. Assume the training dataset is $Z=\left\{\left(x_{1},y_{1}),\cdots (x_{i},y_{i}),\cdots (x_{n},y_{n}\right)\right\}$, where $x_{i}\in \chi =R^{n}$, $y_{i}\in$ψ={+1,−1}, and i=1,2,⋯,n, then $x_{i}$ is the *i*th input feature vector and $y_{i}$ is the category label of $x_{i}$. By introducing a slack variable $\xi_{i}$, the learning problem of linear indivisible SVM can be described as a soft interval maximization problem [42]:

(9) $$\begin{array}{l} \underset{w,b,\xi }{\min}\;\frac{1}{2}\left\|w\right\|{\;}^{2}+C\sum_{i=1}^{n}\xi_{i}\\ s.t.\begin{array}{cc} \; & y_{i}(w\cdot x_{i}-b)\geq 1- \end{array}\xi_{i}\begin{array}{cc} & \forall (x_{i},y_{i})\in Z \end{array}\\ \begin{array}{cc} \; & \xi_{i}\geq 0 \end{array},\;i=1,2,\cdots ,N \end{array}$$

where *w* and *b* are optimization parameters, $\xi_{i}$ is a slack variable, and *C* is a penalty factor. By continuously adjusting *w* and *b*, the objective function is minimized. Finally, the maximum separation hyperplane is obtained [42]. Solving the dual problem of Equation (9) and selecting the suitable kernel function $K(x_{i}\cdot x_{j})$ and the penalty factor *C* (*C* > 0), the separation hyperplane that solves the nonlinearity can be obtained as:

(10) $$\sum_{i=1}^{n}\alpha_{i}^{*}y_{i}K(x\cdot x_{i})+b^{*}=0$$

where $\alpha_{i}^{*}$ and $b^{*}$ are the optimal solutions. The classification decision function can be written as

(11) $$f(x)=sign\left(\sum_{i=1}^{n}\alpha_{i}^{*}y_{i}K(x\cdot x_{i})+b^{*}\right)$$

The kernel function K(x⋅z) in Equation (11) is the core technology of a nonlinear SVM. This paper uses the Gaussian radial basis function (RBF) as the kernel function of a nonlinear SVM in a CNN-SVM model. The mathematical expression of RBF is defined as [42]:

(12) $$K(x_{i},x_{j})=\exp\left(-\frac{{\left\|x_{i}-x_{j}\right\|}^{2}}{2\sigma^{2}}\right)$$

Finally, combining Equations (10) and (11), the classification decision function using the RBF kernel function can be written as

(13) $$f(x)=sign\left(\sum_{i=1}^{n}\alpha_{i}^{*}y_{i}\exp\left(-\frac{{\left\|x_{i}-x_{j}\right\|}^{2}}{2\sigma^{2}}\right)+b^{*}\right)$$

**Figure 7:**
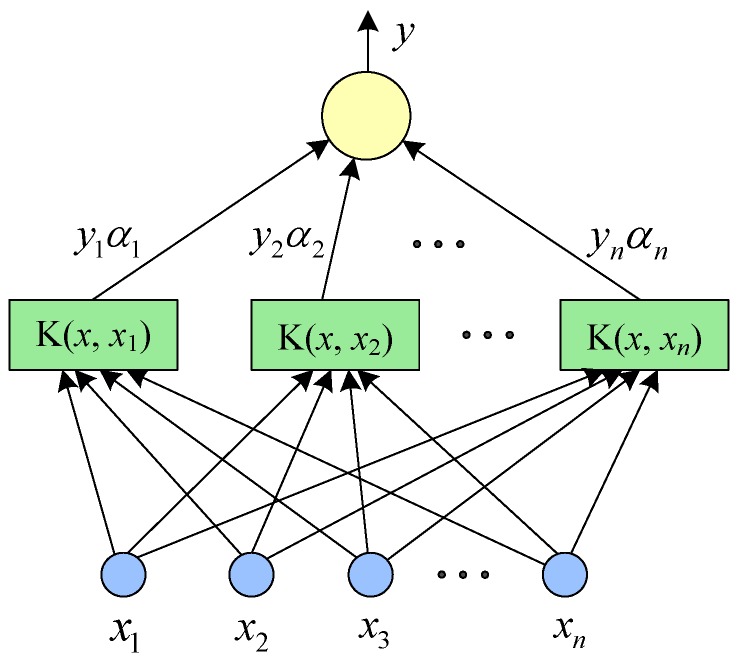
The model structure of SVM.

The SVM algorithm was originally designed for the 2-classification problem. When dealing with multiple types of problems, it is necessary to construct a suitable multi-class classifier. The model structure of SVM is shown in Figure 7. The constructing method of multi-class classifiers can be seen in Reference [45]. 

### 3.2. Intelligent Fault Diagnosis Based on Improved CNN-SVM Method

**Figure 8:**
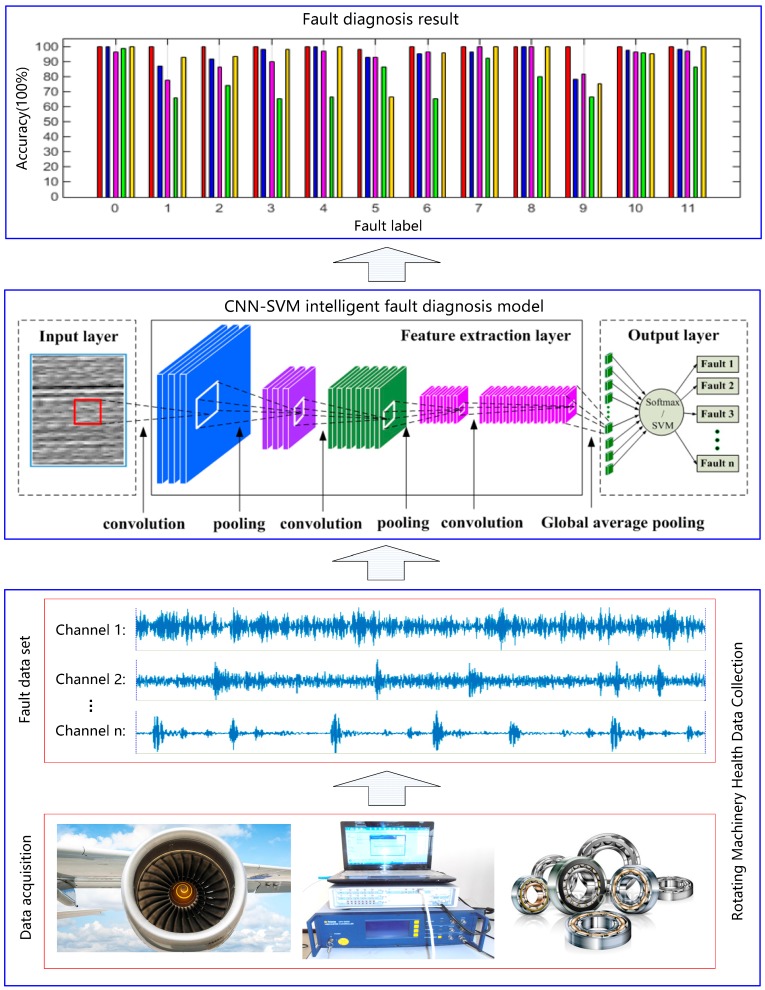
The framework of the proposed CNN-SVM adaptive intelligent fault diagnosis method.

In this paper, a novel method called the improved global average pooling CNN-SVM method is proposed for the intelligent diagnosis of incipient faults in rotating machinery. This method can be divided into three main steps. Firstly, the multichannel raw fault data of the temporal and spatial from multiple sensors is directly input into the improved CNN-Softmax model for the training of a CNN model by the back-propagation algorithm. Secondly, the trained CNN are used as feature extractors to extract representative features of new fault raw data. Finally, the extracted sparse representative feature vectors are input into the SVM used for fault classification. The framework structure of the improved CNN-SVM model is shown in Figure 8. The proposed method is more effective and rapid than the traditional CNN. The proposed CNN-SVM method need not any manual feature extraction and signal processing operations on the raw data during the whole fault diagnosis process. The multichannel raw fault data can be directly input into the proposed model, and the fault classification result is automatically output. The proposed method which has an end-to-end algorithm structure has a good operability and versatility. 

**Figure 9:**
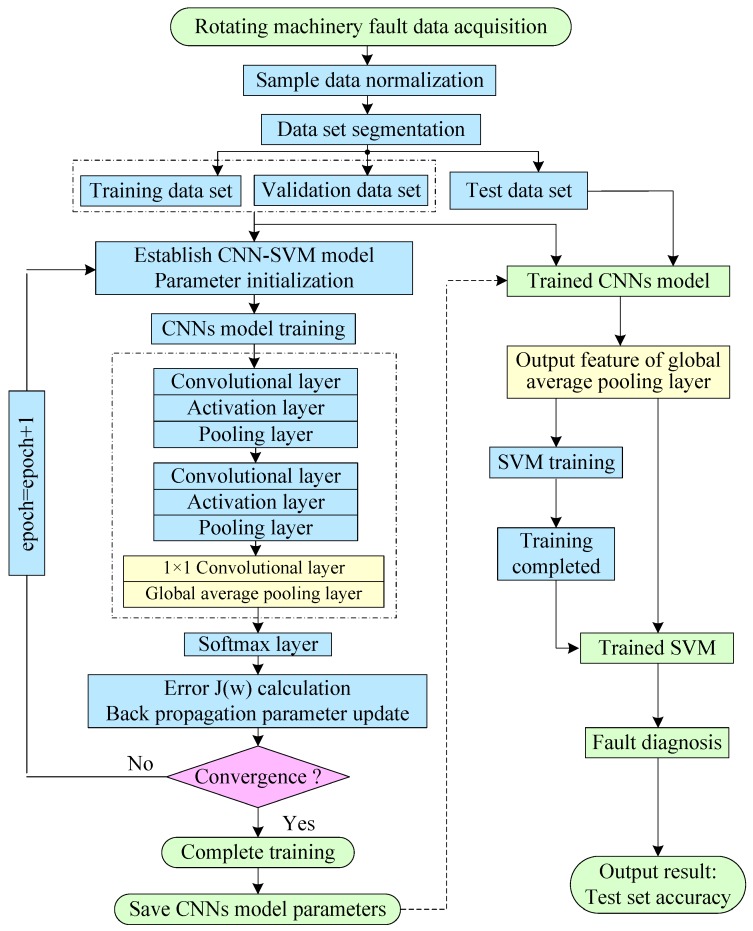
The flow chart of the CNN-SVM intelligent fault diagnosis algorithm.

In Figure 8, the application object of the proposed method includes aircraft engines, generators, electromotor, and other common rotating parts in machinery. The fault types contain the common faults in rotating machinery such as the mechanical wear, fracture, deformation, and rotor eccentricity imbalance, etc. The fault signal data include common monitoring signals such as vibration, noise, current, and pressure, etc. In Figure 8, there are three modules in the proposed CNN-SVM fault diagnosis framework. The lowest layer is the data acquisition module of the rotating machinery. The top layer is the resulting output module of the fault diagnosis. The middle layer is the CNN-SVM intelligent algorithm module. The proposed method can get rid of the dependence on manual feature extraction and can overcome the limitations of traditional methods relying on expert experience. The basic flow chart of the CNN-SVM intelligent fault diagnosis algorithm is shown in Figure 9. 

In Figure 9, there are two stages: the training process and the test process. In the training stage of the CNN-SVM model, there are mainly three steps: Firstly, the Softmax function is combined with multilayer CNN model, and the training of CNN model parameter is completed. The error between the predicted result of the CNN model and the actual label of sample is calculated. The loss function J(w) is minimized by the error back-propagation algorithm using the Softmax function. Secondly, the representative feature of the raw input data is automatically extracted by using the trained CNN model. Thirdly, the extracted low-dimensional representative feature data and their corresponding tags are input into the SVM model to complete the training of SVM. 

In the test stage of the CNN-SVM model, there are mainly three steps: Firstly, the trained CNN model is used to extract features from new fault data. Also, the sparse low-dimensional representative feature vectors of new fault input data are obtained. Secondly, the extracted low-dimensional feature data are input into the trained SVM classifier to complete the final fault classification. Finally, the result of the fault diagnosis and time record is automatically output. 

### 3.3. Deep Learning Training Skills 

Due to the inconsistency of the raw fault dataset and the lack of training samples, the efficiency of trained DNN model is poor. In order to improve the accuracy of fault diagnosis and to prevent model over-fitting, deep learning training skills are usually used in the model training stage [4]. After long-term practice and summarization, some effective deep learning skills including batch normalization, data augmentation, Dropout, mini-batch, and adaptive variable learning rate are introduced [2,4,33]. 

(1) Batch normalization 

Batch normalization is one of the most common training techniques in deep learning; the main role is to process the output data to the same magnitude standard data [46]. In the CNN, the model weight parameters are updated by a chain derivation method of backward propagating. The cost error J(w) is propagated layer by layer from the end of the network to the front end. The gradient values of the trainable parameters are accumulated during the update process. The gradient explosion or gradient disappearance will occur when multiple gradient values greater than 1 or less than 1 are multiplied in the deep neural network [23]. To solve this problem, the batch normalization method is used for the output data of each layer. Batch normalization techniques can adjust the output value of forward propagation having the same distribution to the largest extent. By batch normalization, the sample distribution characteristics within the same layer are preserved while the distribution gap between layers is eliminated. Therefore, the distribution of the data samples referenced in the backward propagating calculation is consistent with the distribution in the forward propagating calculation [5]. Therefore, the weight adjustment of a model is more reasonable during the error back propagation process [46]. The batch normalization function can be defined as:

(14) $$x_{i}^{\prime }=\frac{x_{i}-\bar{x}}{x_{\mathrm{var}}}=\frac{x_{i}-\frac{1}{n}\sum_{j=1}^{n}x_{j}}{\sqrt{\frac{1}{n}\sum_{j=1}^{n}{\left(x_{j}-\bar{x}\right)}^{2}}}$$

where $\bar{x}$ is the average value of n input data *x* and $x_{\mathrm{var}}$ is the variance value of n input data *x*. 

(2) Data augmentation 

**Figure 10:**
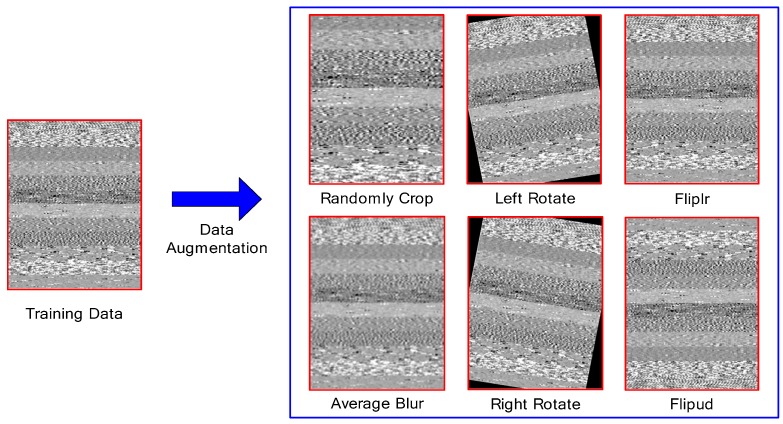
The data augmentation operations of a two-dimensional feature map of a vibration signal.

CNN is suitable for processing large data sample sets. The over-fitting problem is easily generated in a CNN when the training samples are insufficient [4]. The over-fitting problem will result in a good fitting performance of the trained model to the training dataset but a poor generalization ability to the new fault data [23]. Data augmentation technology is an effective method to solve the sample shortage in deep learning. It expands the original dataset and improves the generalization ability of the model by a series of small-scale random images transformations to the original feature image. It has been widely used in image recognition [47]. The common data augmentation operation includes random cropping, rotation, flipping, etc. For the one-dimensional time series vibration signal fault data of the rotating machinery, it can be reconstructed into a 2-D feature map firstly, and then, the data augmentation operations are applied according to the image processing method, as shown in Figure 10. In Figure 10, the left side is the original training data feature map. The right side is six data augmentation operations including random cropping, left-right flipping, left rotation, average blurring, up-down flipping, and right rotation of the original data. 

(3) Dropout technology

**Figure 11:**
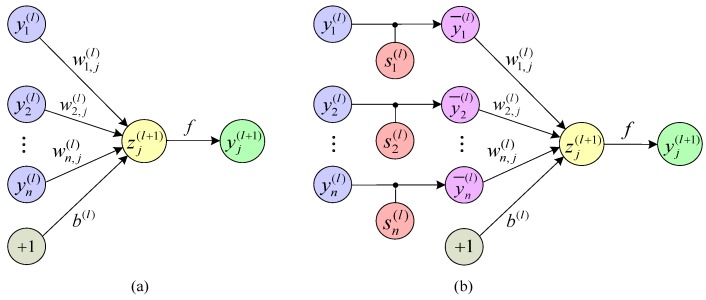
A comparison of the normal and dropout networks: (**a**) A standard network and (**b**) a Dropout network.

The dropout technology is a very simple and effective method to prevent the over-fitting of the model and was proposed by Srivastava and Hinton [48]. Because the fault data collected in most engineering practices are not absolutely pure, it contains some abnormal data [23]. As the fault dataset contain abnormal data, the CNN model has difficultly completely separating the data of different categories. Because the CNN model learns those abnormal data as normal data, this results in the trained model being distorted and over-fit [42]. The over-fitting model has a high accuracy on the training set but a low accuracy on the test set and other generalization applications. In actuality, the abnormal data are usually much less than the normal data in a sample. Therefore, the abnormal data have less occurrence probabilities than the normal data in the same situation. The main idea of Dropout is to reduce the impact of such abnormal data on the model [46]. The dropout technology abandons a certain proportion of neurons in the network randomly and temporarily [2]. This operation can effectively reduce the occurrence probability of abnormal data and the impact on the model. Because the neurons are randomly discarded, each mini-batch is trained in a different network. Therefore, the total training parameter quantity of the model is unchanged [23]. The dropout technique is only used for the training process of the model, and all neurons of the model still work during the test stage. After using the dropout skill, each unit of the neural network in the training process needs to add a probability process, as shown in Figure 11 [48]. 

The expression of a standard network without a dropout operation (Figure 11a) can be written as:

(15) $$\left\{ \begin{array}{l} z_{j}^{\left(l+1\right)}=\sum_{i=1}^{n}w_{i,j}^{\left(l\right)}y_{i}^{\left(l\right)}+b_{}^{\left(l\right)}\\ y_{j}^{\left(l+1\right)}=f(z_{j}^{\left(l+1\right)}) \end{array}\right.$$

where $y_{i}^{\left(l\right)}$ represents the input value of the *i*th neuron in the *l*th layer; $y_{j}^{\left(l+1\right)}$ represents the final output value of the *j*th neuron of the (*l* + 1)th layer; $z_{j}^{\left(l+1\right)}$ represents the linear combined output value of the *j*th neuron of the (*l* + 1)th layer; *i* and *j* represent the *i*th and *j*th neurons, respectively; *l* and *l*+1 represent the *l*th layer and the (*l* + 1)th layer of the model; $w_{i,j}^{\left(l\right)}$ represents the weight value between the *i*th neurons of the *l*th layer and the *j*th neuron of the (*l*+1)th layer; $b_{}^{\left(l\right)}$ is the bias of the *l*th layer; and f(⋅) is the activation function. 

The feed-forward operation calculation of a Dropout network (Figure 11b) can be written as:

(16) $$\left\{ \begin{array}{l} s_{i}^{\left(l\right)}\;∼Bernoulli(p)\\ {\bar{y}}_{i}^{\left(l\right)}=s_{i}^{\left(l\right)}*y_{i}^{\left(l\right)}\\ z_{j}^{\left(l+1\right)}=\sum_{i=1}^{n}w_{i,j}^{\left(l\right)}{\bar{y}}_{i}^{\left(l\right)}+b_{}^{\left(l\right)}\\ y_{j}^{\left(l+1\right)}=f(z_{j}^{\left(l+1\right)}) \end{array}\right.$$

where $s_{i}^{\left(l\right)}$ is the vector of Bernoulli random variables which has a probability *p*. When $s_{i}^{\left(l\right)}$ = 0, it means that the neuron is discarded. When $s_{i}^{\left(l\right)}$ = 1, it means that the neuron is retained. 

(4) Mini-batch adaptive learning rate optimization algorithm

The gradient descent algorithm is used for minimizing the loss function J(w) according to the rule of error back propagation [24,35]. The weight and bias of the model’s trainable parameters are updated in the training process of the CNN model. In deep learning, according to the ratio of training samples accounting for the total sample dataset in each iterative calculation, the training methods can be divided into the following three types: Batch gradient descent (all samples), Stochastic gradient descent (single sample), and Mini-batch gradient descent (mini-batch samples) [23]. The most common training mode in deep learning is the mini-batch [33]. The mini-batch avoids the slow convergence of the model, and it reduces the gradient disappearance and convergence instability [23]. Therefore, in this paper, the mini-batch gradient descent is applied. 

The learning rate is an important indicator of model training. It determines the amplitude magnitude of the model parameter update. When the learning rate is too high, the training speed will increase, but the result will be difficult to converge to a minimum or to fluctuate around the minimum. If the learning rate is too low, the training accuracy of each epoch iteration will be improved, but the convergence speed of the model is too slow and easy to fall into the local optimum [39]. In this paper, the Adam [49] optimization algorithm is applied to train the CNN model. In the Adam algorithm, the learning rate can automatically adjust the learning step size according to the local error surface of the mini-batch sample. The learning rate increases when the error approaches the target in a reduced trend. The learning rate decreases when the error exceeds a certain multiple of the previous error in an increasing way. At the same time, the update operation that increases the error is deleted. The learning rate adjustment method can be described as follows:

(17) $$\eta (k+1)=\{\begin{array}{l} k_{inc}\;\eta (k),\;E(k+1)<E(k)\\ k_{dec}\;\eta (k),\;E(k+1)>(1+c)E(k)\\ \eta (k),\;E(k+1)=E(k) \end{array}$$

where η(k) represents the learning rate of the current training, η(k+1) indicates the next updated learning rate, $k_{inc}$ is the learning rate increment factor, $k_{inc}>1$, $k_{dec}$ is the learning rate reduction factor, $k_{dec}<1$, E(k) represents the current error value, E(k+1) represents the error value of the next iteration after the update, *c* is a constant, and c>0. The basic training process of the Adam adaptive learning rate optimization algorithm can be seen in Reference [49].

## 4. Experimental Verification Based on Multichannel Vibration Signals 

In order to evaluate and verify the effectiveness and accuracy of the proposed CNN-SVM method for the intelligent diagnosis, in this paper, the proposed method is applied to diagnose the experimental vibration signals data of rolling bearing and is compared with traditional intelligent diagnosis methods. In general, the vibration signals are the most common monitoring indicators for rotating machinery, it is easy to acquire by using the accelerometers [50]. In order to increase the diagnosis accuracy and reliability, in this paper, the multichannel fusion fault dataset is established by collecting the signal data of multiple vibration sensors. The vibration signals from different working conditions and different sensors can be aligned into a 3-D matrix as the input of the CNN-SVM model. The temporal and spatial information of the bearing fault data is integrated. The results confirm that the proposed method is more effective than other existing intelligent methods such as SVM, KNN, BPNN, DBPN, and traditional CNN. 

### 4.1. Multichannel Fusion Fault Dataset of Rolling Bearing 

**Figure 12:**
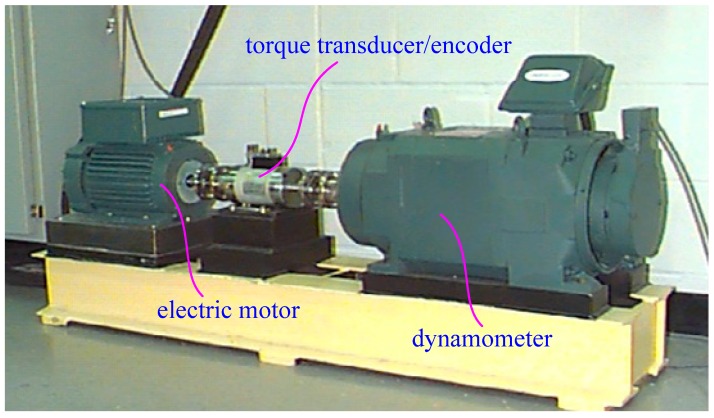
The rolling bearing fault data acquisition experimental bench.

Rolling bearings are the most important rotating shore components in rotating machinery, and their running accuracy and safety reliability directly affect the overall performance of rotating machinery [3]. In a practical work, rolling bearings have various faults due to an alternating load, thermal fatigue, and mechanical wear under the complicated and variable working conditions at high speeds for a long time operation. In this study, the experimental vibration data of rolling bearing are from the motor experimental bench of the Electrical Engineering Laboratory of Case Western Reserve University (CWRU) [51], as shown in Figure 12. The experimental device uses a 2-horsepower three-phase electric motor (left) produced by Reliance of India as the power source [51]. A dynamometer (right) is used to generate the experimental load. A torque transducer/encoder (middle) is mounted between the electric motor and the dynamometer for measuring speed and torque. The test bearing is used for supporting the motor shaft. The vibration signal data of the drive end bearing and the fan end bearing are collected by using a 16 channel DAT recorder and 2 accelerometers [51]. The 2 accelerometers are respectively mounted at the 12 o’clock positions at both the drive end and fan end of the motor housing by a magnetic seat. 

The bearing geometry specifications of the drive end and fan end are listed in the Table 2 [51]. The bearings on the drive end and fan end are deep groove ball bearings, and the modes are 6205-2RS JEM and 6203-2RS JEM respectively, produced by SKF, Sweden. By analyzing the failure mechanism of rolling bearings, it is known that the failure types mainly include the pitting, wear, gluing, and plastic deformation. Also, the location where the fault occurs is mainly distributed in the rolling elements, inner ring, and outer ring of the bearing. Pitting is usually the most common and earliest failure type, showing the tiny pitting and pits on the rolling element, inner ring, and outer ring, and the pitting size gradually increases with time [7]. In this experiment, the single point faults are introduced to simulate the pitting failures of rolling bearings. The pits with different sizes are machined on the test bearings by using electro-discharge processing methods. Pits with fault diameters of 0.007 inches, 0.014 inches, and 0.021 inches are machined on the rolling element, the inner ring, and the outer ring of the bearing, respectively. The difference in fault diameters represents the evolution process of a pitting fault gradually increasing from micro-fault to significant fault. To investigate the fault characteristics under different working conditions, the vibration signal data of the rolling bearing are collected under four load conditions: 0 horsepower (1797 r/min), 1 horsepower (1772 r/min), and 2 horsepower (1750 r/min). The sampling frequency of all data is 12 KHz, and the sampling time is about 10 s. 

Based on the above work, a multichannel fault dataset of rolling bearings under the multiple working conditions are established, as shown in Table 3. The detailed description of the fault dataset establishment process is as follows: 

Firstly, the vibration signal data of different bearing states under different working conditions are collected. The established fault dataset contains 10 bearing health statuses, including one normal state and nine fault types. Nine fault states correspond to three fault locations and three fault severities, respectively. The three fault locations are rolling ball, inner raceway, and outer ring. The three fault levels are the incipient micro-fault, the less obvious moderate fault, and the obvious significant fault corresponding to the fault size from 0.007–0.021 inches. 

Secondly, each health state contains 2 channel vibration data, one from the drive end vibration sensor and the other from the fan end vibration sensor. Each vibration sensor collects 10-second vibration signals and obtain 12,000 data points per second under the sampling frequency of 12 KHz. Therefore, there are a total of 120,000 vibration data points in each channel. 

**Figure 13:**
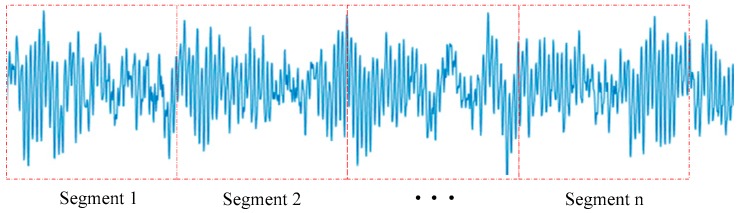
The vibration signal data segmentation.

Thirdly, the vibration signal of each channel is split into multigroups of equal length data segments. On the one hand, data segmentation could prevent inputting 120,000 data points into the CNN-SVM model at one time and can cause computer memory overflow. On the other hand, data segmentation can create more samples to facilitate training and testing the CNN-SVM model. The process of dividing signals into segments is shown in Figure 13. In this experiment, as the speed range of the four loads is 1750–1797 r/min, each sensor can collect 400–412 signal points (12000 × 60/1797 ≈ 400) under the per revolution of the motor shaft. In order to ensure the confidence of the fault data, in Figure 13, the length of each data segment is defined as 500 sample points. So, each fault sample is a 500 × 2 data. The first 100,000 sample points are intercepted from the original 120,000 sample points. Therefore, each health state contains 200 500 × 2 samples.

Fourthly, the vibration signals of the same healthy state under multiple load conditions are fused. The multichannel fault dataset under the multiple working conditions is shown in Table 3. 

In generally, rolling bearings often need to bear multiple working conditions with alternating variable loads in actual work. The same fault will produce different magnitudes of vibration shock signals under different loads. Therefore, the fault diagnosis model should be able to accurately identify each fault type of bearings under different load. It is noted that the same load will produce different magnitude impact forces under different fault sizes, but the frequency of the shock vibration signal is the same. As different loads have different rotational speed, the magnitude and frequency of shock vibration signals of the same fault types under different loads are different. In order to further use the monitoring data under the various working conditions to accurately diagnosis the fault types of bearing, in this paper, the multiple working conditions that compound the fault dataset of a rolling bearing is established by combining the vibration data under the three loads including 0 horsepower, 1 horsepower, and 2 horsepower. There are 3 load conditions, so each health state contains 200 × 3 = 600 samples. Finally, the established composite dataset has a total of 6,000 samples, and the total number of vibration data points is 6 million.

**Figure 14:**
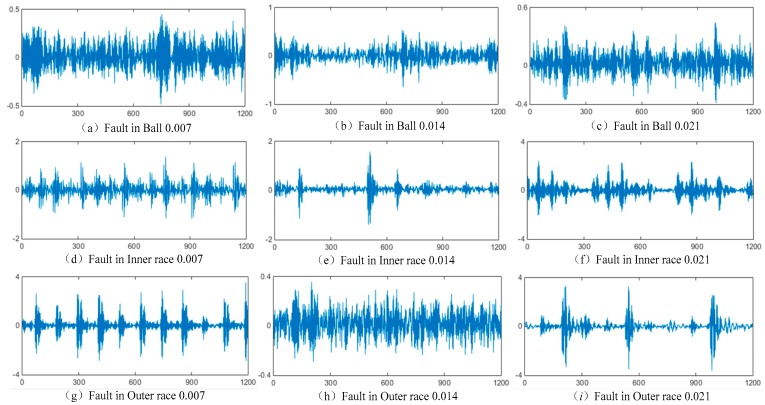
The vibration signal time-domain waveforms of the drive-end rolling bearing under the 0-horsepower load.

Figure 14 shows that the vibration data time-domain waveforms of the 9 fault types of the drive end rolling bearing are collected under the 0 horsepower load. Figure 14a–i are the waveforms of 1 to 9 fault types listed in Table 3, respectively. Due to the limited space of the article, the data descriptions and waveform of other loads are not listed here. 

### 4.2. Fault Data Processing 

In order to better train the CNN-SVM model, the necessary data processing is performed on the rolling bearing fault dataset shown in Table 3. It mainly includes the input data format reconstruction, standardized input data, and dataset partition. The details are as follows: 

(1) Input data format reconstruction 

**Figure 15:**
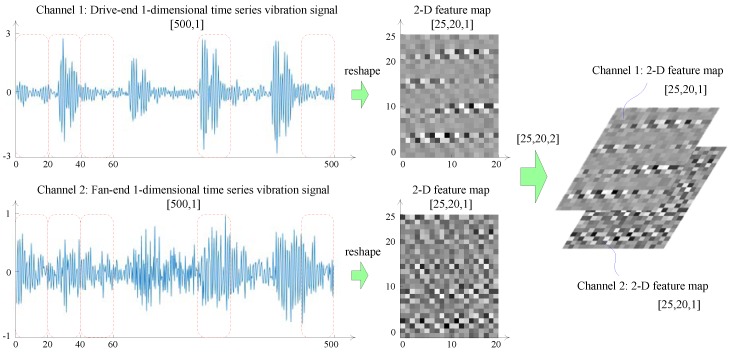
The data reconstruction process of a 2-channel input feature map.

In Table 3, each sample is a 2-channel one-dimensional time series data. According to the data reconstruction method shown in Figure 6, the two-channel one-dimensional time series original data (500, 2) are reconstructed into a 3-dimensional input feature map form (25, 20, 2), where 2 represents 2 channels, 25 represents the height of feature map, and 20 represents the width of feature map. The data reconstruction process is shown in Figure 15. 

(2) Input feature map data standardization 

Data standardization is a common input data processing method. To eliminate the magnitude difference between different data, data standardization converts all input data into values between [0,1]. Avoid the network prediction errors that are too large due to excessive differences in the magnitude of the input data. In this paper, the input samples are standardized by the maximum–minimum mean method, and the mathematical expression is described as follows:

(18) $$X=\left\{x_{i}\right\}=\frac{x_{i}-x_{\min}}{x_{\max}-x_{\min}}$$

where $x_{\max}$ represents the maximum value in the input sample, $x_{\min}$ represents the minimum value in the sample, X is the result after standardization, and the value range is between 0 and 1. 

(3) Dataset division 

All the samples of each type fault after standardization are randomly divided into a training stage dataset and a test stage dataset. The ratios are 70% and 30%, respectively [4]. Then, 20% of the samples of the training stage dataset are randomly taken as the verification dataset. The description of the dataset partition is shown in Table 4. 

As shown in Table 4, there are 200 × 3 samples in each type of fault, where 3 represents 3 load types. In order to ensure dataset equalization in the operation of dataset dividing, firstly, randomly take 70% (140 samples) from the 200 samples under each load as the training stage dataset, and the remaining 30% (60 samples) is used as the test dataset. Then, randomly take 20% samples (140 × 0.2 = 28) from the training stage dataset as the verification dataset and the remaining 80% (112 samples) as the training dataset. Finally, the training dataset, verification dataset, and testing dataset of each health state under three loads are respectively combined into a total training set (112 × 3 = 336 samples), a total verification set (28 × 3 = 84 samples), and a total testing set (60 × 3 = 180 samples). Therefore, the total number of training datasets, verification datasets, and test datasets corresponding to the 10 bearing health states are 112 × 3 × 10 = 3360, 28 × 3 × 10 = 840, and 60 × 3 × 10 = 1800, respectively.

In this paper, the training dataset is used for training the CNN-SVM model. During the training process, the model hyper-parameter is compared and selected according to the verification set precision curve. Simultaneously, the verification dataset is used for verifying the accuracy of the trained CNN-SVM model. When the error on the verification set is larger than the error on the training set, it indicates that the model may be over-fit, and the model training is stopped in time [2,4]. Usually the training process takes a relatively long time. Finally, the test dataset is used for the final fault classification and accuracy assessment of the model. 

### 4.3. Hyper-Parameters Selection of CNN Model 

The CNN as the feature extractor of the CNN-SVM model has a great influence on the final fault diagnosis result of the CNN-SVM model. In the process of constructing the CNN model, selecting the appropriate CNN model hyper-parameters can effectively improve the fault diagnosis accuracy, training speed, and test speed of the CNN-SVM model. In general, the hyper-parameters that are important influence the performance of the CNN model and mainly include activation functions, optimizers, learning rates, convolution kernels, and pooling kernels [32,39]. To investigate the influence of different hyper-parameter settings on the performance of the CNN model, a reference CNN model is established by multiple repeated trials. The established multichannel fault composite dataset under a 0–2 hp load condition are used for the training of the reference model. The impact of different indicators on the model performance is investigated by changing the different hyper-parameters of this benchmark CNN model. The experiment results are used to guide the design of the CNN-SVM model. This reference model includes a 2-layer CNN feature extraction module, a 2-layer fully connected network, and a Softmax classifier. The hyper-parameters of the benchmark model are listed in Table 5. The model uses the Adam adaptive optimization algorithm and the cross-entropy loss function, and the learning rate is adaptively adjusted. The experiment is completed on the computer with Intel i5-7500 CPU, 16GB memory, and NVDIA 1050 Ti GPU. 

The calculation method of the parameter quantity in each network layer in Table 5 is as follows:

(19) $$\left\{ \begin{array}{l} {\mathrm{CNN}}_{\mathrm{num}}=K_{num}\times k_{height}\times k_{width}\times I_{num}+B_{num}\\ {\mathrm{FCN}}_{\mathrm{num}}=N_{input}\times N_{output}+B_{hidden} \end{array}\right.$$

where ${\mathrm{CNN}}_{\mathrm{num}}$ is the parameter quantity of the convolution layer, ${\mathrm{FCN}}_{\mathrm{num}}$ is the parameter quantity of the fully connected layer, $K_{num}$ is the quantity of convolution kernels (the number of output channels), $k_{height}\times k_{width}$ represents the height × width of the convolution kernel, $I_{num}$ is the number of input data channels, $B_{num}$ is the number of biases of the convolutional layer, $N_{input}\times N_{output}$ represents the product of the number of neuron nodes between two adjacent layers in the fully connected layer, and $B_{hidden}$ is the bias of the hidden layer. 

(1) Selection of activation function 

The activation function has a great influence on the training of the CNN network. Usually, the activation function is selected according to working experience [3]. However, this method is too casual and not convincing. In this paper, the dataset of Table 4 is calculated according to the CNN model setting of Table 5. The iterations epoch is 100. The effects of the three most commonly used activation functions are compared by using the reference CNN model. The activation functions include Sigmoid, Tanh, and Relu. The experimental calculation results are listed in Table 6. 

In Table 6, the two convolutional layers use the same activation function. By comparing the 9 experimental results in Table 6, the following conclusions can be obtained: in terms of accuracy, experiment 4 has the highest training accuracy rate. Comparing experiments 1, 2, and 3, it shows that experiment 3 has the worst performance. The results of experiments 3, 6, and 7 are very poor, indicating that the sigmoid function has the worst effect on the convolutional layer. Comparing experiment 2, 4, and 7, the effect of the Relu function is the best in the convolutional layer. Comparing experiments 1, 4, and 5, the effect of the Tanh function is the best in the fully connected layer. As can be seen from the calculation time, the training and testing time of Experiment 1 is the shortest, indicating that the Relu function is faster. The sigmoid function converges slowly compared with the other two activation functions. Therefore, this paper uses the Relu function as the activation function of CNN in the CNN-SVM model and uses the Tanh function as the activation function of the fully connected layer in the subsequent experiments. 

(2) Selection of optimization algorithm 

**Figure 16:**
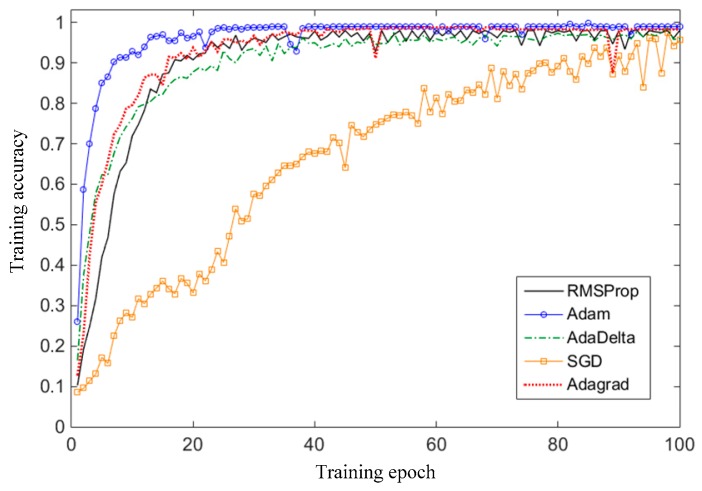
The performance curves comparing the charts of five optimization algorithms.

For different deep learning models and classification tasks, choosing the appropriate optimization algorithm plays an important role in improving the training speed and classification accuracy of the model. In this experiment, the five most commonly used optimization algorithm including Steepest Gradient Descent (SGD), Adagrad, Adadelta, RMSProp, and Adam optimization algorithm are tested and compared. In this optimization algorithm selection experiment, the reference CNN model parameter settings in Table 5 are used. The Relu activation function is used for the convolutional layer, and the Tanh activation function is used for the full connection layer. The experimental results are listed in Table 7. Figure 16 shows the training accuracy curves of the five optimization algorithms in the first 100 iterations. It can be clearly seen from Table 7 and Figure 16 that the performance of the Adam adaptive optimizer is significantly better than other four optimizers. Especially in the first 30 epochs of iterative calculations, the training accuracy of the Adam optimization algorithm rises quickly and the convergence in the later stage is more stable. In terms of the training accuracy, the Adam optimizer has the best performance and the SGD effect is the worst in this experiment. In the Adam algorithm, the learning rate can be automatically adjusted according to the local error surface of the mini-batch sample. In terms of training time, the five optimization algorithms have no obvious difference. The RMSProp optimization algorithm has the least training time. However, Adam has the best performance in terms of test time. In summary, this paper uses the Adam adaptive algorithm as the training optimizer of the subsequent CNN-SVM method.

(3) Selection of the mini-batch samples number 

Deep learning usually uses mini-batch methods to train samples. The different number of samples per mini-batch has a significant impact on the performance of the model. In order to study the effect of the sample number per mini-batch on the performance of the model, the experiment is conducted by setting different values of mini-batch. Usually, the value of mini-batch uses a power of 2 to get less runtime, which ranges from 32 to 256 [23]. In this experiment, the reference model parameter settings in Table 5 are used. The Adam optimizer is applied. The Relu activation function is used for the convolutional layer. The Tanh activation function is used for the full connection layer. The experimental results are listed in Table 8. 

It can be seen from Table 8 that the best training accuracy can be obtained when mini-batch is set to 64. When mini-batch is 64, the accuracy is relatively higher than others and the training time is not too long. When mini-batch is 512, the total training time is the shortest but the accuracy is only 0.8729. When mini-batch is 1, it means there is only one sample per batch and it is easy to fall into a local optimum. Although the speed of processing a single batch is the highest, the model needs more batches to complete the calculation of all samples. Therefore, the final training time of the model is the longest, up to 4286.88 s, and the accuracy is merely 0.9585. Therefore, in the subsequent training of CNN-SVM models, the mini-batch is set to 64. 

(4) Selection of the CNN model structure 

**Figure 17:**
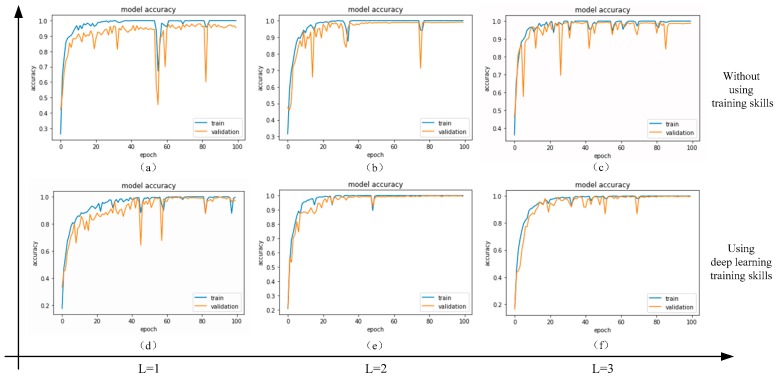
The training process of different CNN models.

To investigate the impact of a different CNN model structure on accuracy performance, in this experiment, three CNN models structure are designed including a one-layer CNN, a two-layer CNN, and a three-layer CNN structure. Each CNN model structure is divided into two cases: without using deep learning training skills and using deep learning training skills. The training skills include data augmentation and Dropout. The super-parameters of different CNN models are listed in Table 9. The Adam optimization algorithm is applied. The learning rate is automatically adjusted. The Relu activation function is used for the convolutional layer. The Tanh activation function is used for the full connection layer. The mini-batch is set to 64. The iterations epoch is 100. The diagnosis results are listed in Table 10. The accuracy curves of different CNN model are shown in Figure 17. 

It can be obviously seen from Table 10 and Figure 17 that the two-layer CNN model using the deep learning training skills has the best performance and that the test accuracy is up to 98.99%. The performance of the one-layer CNN model is the worst, and the feature extraction ability is insufficient due to the simple structure. In this experiment, as the sample quantity of the training dataset is less, the model using the three-layer CNN is worse than the two-layer CNN model and easily causes a gradient disappearance and over-fitting of the deep convolution network structure. In addition, the training time and test time of the multilayer model structure is longer than the simple model because the model parameters increase as the number of CNN layers increase. In Figure 17, the dark blue curve represents the accuracy of the training dataset and the orange curve represents the accuracy of the validation dataset. It can be seen from the Figure 17a–c that the validation dataset accuracy curve is lower than the training dataset accuracy curve, which shows that the CNN model has generated over-fitting. Comparing Figure 17a–c with Figure 17d–f, it can be seen that the data augmentation and dropout training skills can effectively reduce the over-fitting of the model and can also improve the accuracy of the model in a small range. 

### 4.4. Fault Diagnosis Results and Evaluate of Proposed CNN-SVM Model

(1) Fault diagnosis results of CNN-SVM algorithm 

According to the results and conclusions of the above research, the CNN-SVM intelligent fault diagnosis model is finally established. The detailed hyper-parameters are listed in Table 11. This model is improved on the basis of the two-layer CNN model shown in Table 5. Firstly, the global average pooling layer is used instead of the fully connected layer structure. Secondly, the dropout technology, data augmentation, and batch normalization skills are applied in the training stage of CNN to prevent the over-fitting problem. The proposed method sets a Dropout layer after each convolutional network. Thirdly, a 1 × 1 transitional convolution layer is set between the 2-layer convolution network and the global average pooling layer. Here, the role of the 1 × 1 transitional convolutional layer is to change only the output dimension of the feature map without changing the feature map shape. For a detailed description of the improved CNN model, refer to Section 3.1 and Figure 5. In this experiment, the fault type of rolling bearing is 10, so the dimension of the transitional convolutional layer is set to 10. The total framework of the proposed CNN-SVM adaptive intelligent fault diagnosis method is shown in Figure 8. 

A comparison of the trainable parameter quantity between the traditional CNN model and proposed CNN-SVM model is listed in Table 12. As can be seen from Table 12, the total parameter amount of the traditional CNN model structure using the fully connected layer is up to 143,978. The total training parameter of the improved CNN-SVM model using the global average pooling layer is only 20,120. Therefore, the improved CNN-SVM model can greatly reduce the amount of training parameters. For the calculation method of the parameter quantity, refer to Equation (19). 

In this experiment, the Adam adaptive learning rate optimization algorithm is used for the training stage of CNN-SVM model. The ReLU activation function is used for the 3 activation layers of the CNN-SVM. The number of mini-batch is set to 64. The epoch of iteration calculation is 100. The main parameter of SVM classifier is set with the penalty coefficient C = 10, kernel function is Gaussian radial basis function (RBF), and slack variable ξ=0.1. Finally, the highest accuracy rate of CNN-SVM on the test set is 99.94%. The diagnostic results are shown in Table 13. 

As can be seen from Table 13, there are two calculation results. This is because the proposed CNN-SVM algorithm consists of two stages: one is the training stage of the model, and the other is the testing stage of the model, as shown in Figure 9. The above one is a combination of CNN and Softmax, using the Softmax function for an error backward propagation to train the CNN model parameters. The below one is to extract the feature of the new fault data by using the trained CNN model as a feature extractor and, then, to input the extracted representative features into the SVM for fault classification. As can be seen from Table 13, in terms of accuracy, the accuracy of CNN+Softmax is 99.12%; the accuracy of CNN+SVM has increased to 99.94%. In terms of time, the training time of the CNN+Softmax model is 73.9293 seconds, and the training time of the SVM is 0.3912 seconds. The total training time of the CNN-SVM model is 73.9293 + 0.4912 = 74.3205 seconds. Therefore, although the proposed CNN-SVM method adds an SVM part based on CNN, the training time does not increase too much. In terms of the test time, as the full connection structure is removed, the proposed method only needs 0.08318 seconds. The test time of the improved CNN-Softmax is 0.08967 seconds, which is also obviously better than the traditional CNN-Softmax shown in Table 6 (Experiment 4). The reduction of the test time is of great significance for the proposed method using to rapid fault diagnosis and online real-time monitoring of faults in the future. 

(2) The evaluate of fault diagnosis results 

In order to further analyze and evaluate the classification performance of the proposed CNN-SVM algorithm, in this paper, the Precision ratio, Recall ratio, and F1-measure are calculated [3]. The mathematical expressions of the three indicators are as follows:

(20) $$\left\{ \begin{array}{l} P=TP/\left(TP+FP\right)\times 100\\ R=TP/\left(TP+FN\right)\times 100\\ F_{1}=2TP/(2TP+FP+FN)\times 100 \end{array}\right.$$

where *P* is precision rate, *R* is recall rate, $F_{1}$ is a harmonic average value of the precision rate and the recall rate, *TP* represents the number of true positive instances, *FP* represents the number of false positive instances, *TP* represents the number of true positive instances, and *FN* represents the number of false negative instances. Table 14 lists the precision rates, recall rates, and $F_{1}$-measure values of finally experimental results (as shown in Table 13) of the proposed CNN-SVM method. As can be seen from Table 14, except for the precision rate of condition 8 being 99.45%, the precision rates of the other conditions are 100%. Therefore, it can be illustrated that the proposed CNN-SVM method has an excellent fault diagnosis performance. 

**Figure 18:**
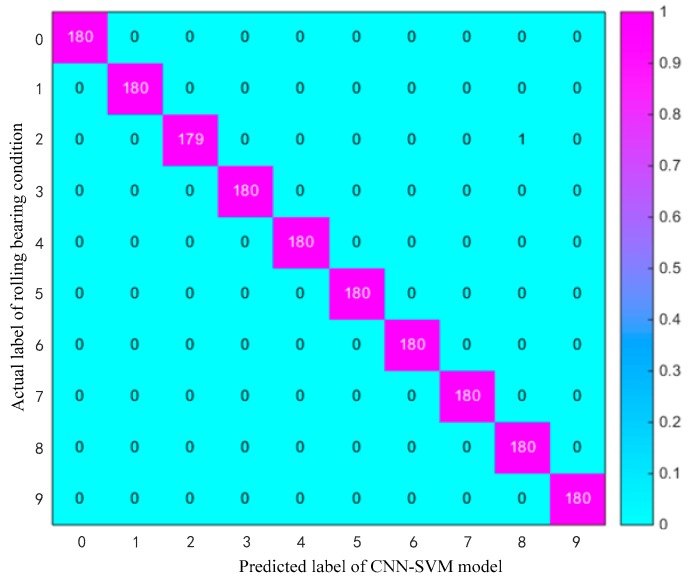
A multi-class confusion matrix of the fault diagnosis results by using the proposed CNN-SVM method.

In order to further assess the performance of the CNN-SVM algorithm to identify incipient micro-faults and the details of fault misjudgment and location, the multi-class confusion matrix [3] is introduced for a detailed quantitative analysis on the fault diagnosis results of the CNN-SVM algorithm. The multi-class confusion matrix comprehensively records the diagnosis classification results and the number of misclassifications of the rolling bearing with different fault types, fault severities, and fault orientations. The multi-class confusion matrix corresponding to the results of Table 14 is shown in Figure 18. 

In Figure 18, the abscissa axis of the multi-class confusion matrix represents the predicted category label of the bearing condition and the ordinate axis represents the actual category label of the bearing condition. The sample number of test datasets for each type of bearing state is 180, and there are 10 types of bearing conditions, as shown in Table 4. The elements on the main diagonal of the multi-class confusion matrix represent the sample number of the correct classification of each bearing health condition. In Figure 18, each column quantifies the precision rate information. Each row quantifies the recall rate information. It can be obviously seen from Figure 18 that the lowest diagnosis result happens in condition 2. Only one of the 1800 test samples on the test set is misclassified by CNN-SVM prediction. The actual label of the misclassified sample is condition 2: Ball (Diameter: 0.014, Depth: 0.011), the predicted category by CNN-SVM model is fault condition 8: Outer Raceway (Diameter: 0.014, Depth: 0.011). Therefore, the fault severity level of both the predicted category and the actual category is the same; only the location where the fault occurs is confused. By checking the computer calculation result data, it can be known that the misclassification occurs in the 0-horsepower working condition. From Figure 18, the diagnosis accuracy of other types of fault is 100%. Especially, the diagnostic accuracy of all incipient micro-faults (condition 1, condition 4, and condition 7) is 100%. The comprehensive fault recognition rate is up to 99.94%. The experimental results show that the proposed CNN-SVM algorithm has a superior recognition ability and a high diagnostic accuracy for incipient faults of rolling bearings. 

(3) Visualizations of Networks 

**Figure 19:**
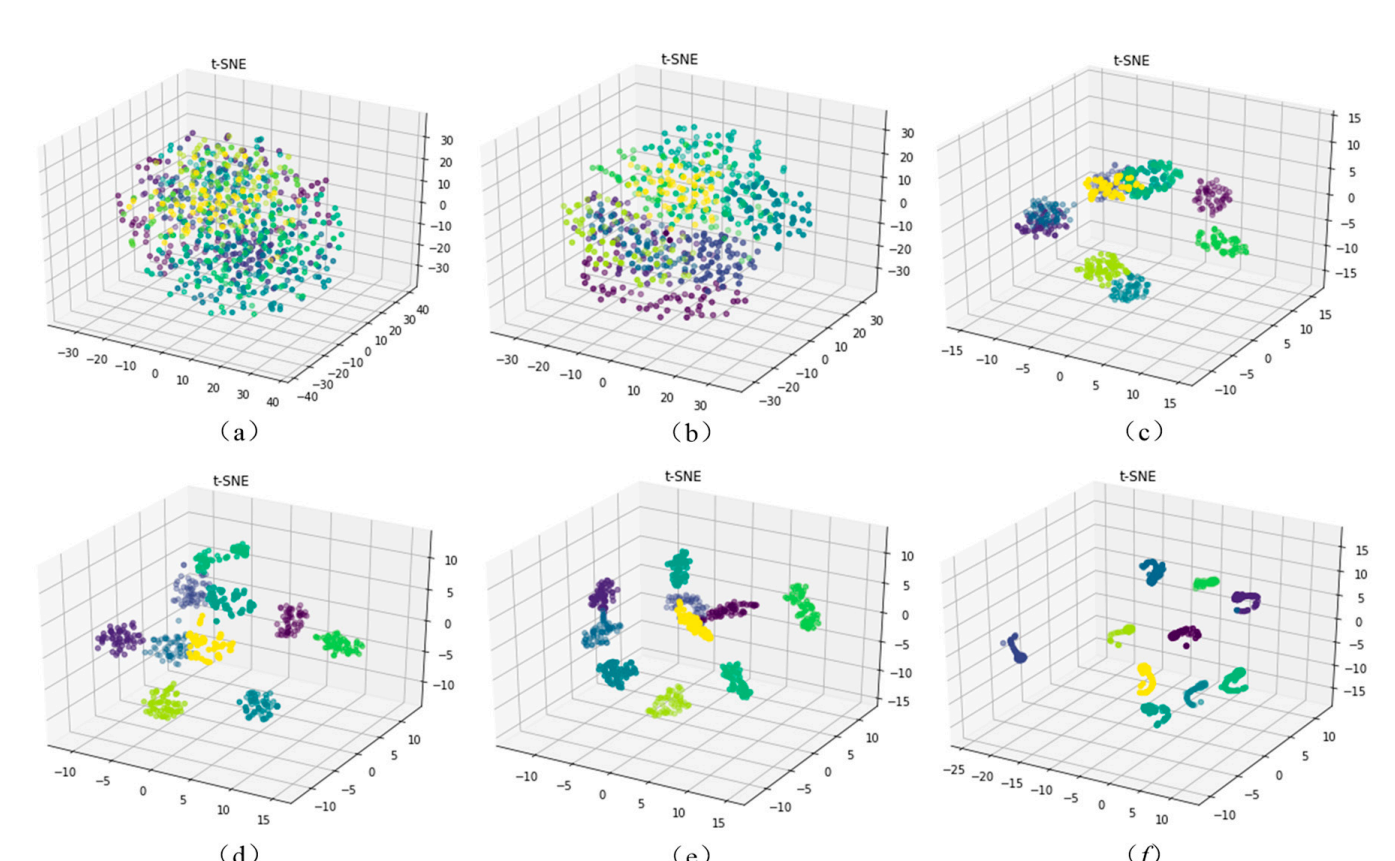
Scatter plots of the feature visualization under test dataset by t-distributed stochastic neighbor embedding (t-SNE): (**a**) The feature distribution of raw data, (**b**) the feature distribution of convolutional layer 1, (**c**) the feature distribution of convolutional layer 2, (**d**) the feature distribution of convolutional layer 3, (**e**) the feature distribution of the global average pooling layer, and (**f**) the feature distribution of the Softmax output layer.

In general, CNN is regard as a black box and the inner operating mechanism of CNN is difficult to understand [2]. In this paper, we try to explore the inner operating process of the proposed CNN-SVM model by the visualization method. In order to further explain the feature extraction ability of each layer of the CNN-SVM model, this paper introduces the most commonly t-distributed stochastic neighbor embedding (t-SNE) [21] method of manifold learning to reduce the dimensions and visualization of the output of each network layer under the test dataset (as shown in Table 4). By mapping the high-dimensional feature vector to the three-dimensional space, the data feature distribution of each layer after a dimensionality reduction by the t-SNE method is shown in Figure 19. 

Figure 19a–f are the raw input data classification visualization, the output of the convolutional layer 1 classification visualization, the output of the convolutional layer 2 classification visualization, the output of the convolutional layer 3 classification visualization, the output of the global average pooling layer classification visualization, and the final output layer classification visualization, respectively. It can be obviously seen from Figure 19a–f that the feature extraction ability of each layer is gradually increased. The 10 health states of the rolling bearing are very confusing in the raw data. Finally, after the Softmax function is calculated, the 10-class bearing health status data is clearly classified. As can be seen from the dark blue point set in the upper right corner of Figure 19f, a few samples are still misclassified, which is consistent with the result of the confusion matrix of Figure 18. The above analysis clearly shows that the trained model has a superior feature extraction ability. 

### 4.5. Comparison with Other Intelligent Algorithms

**Figure 20:**
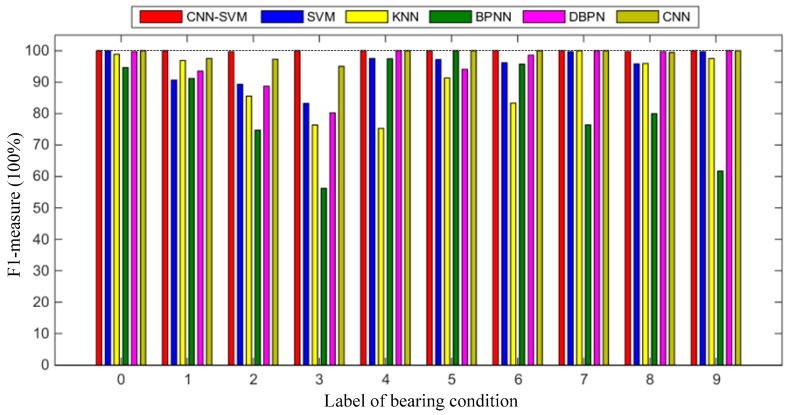
The F1-measures of the bearing data using different methods.

In order to demonstrate the superiority of the proposed CNN-SVM method, four other mainstream traditional intelligent methods are also used to diagnose the same fault dataset (as shown in Table 3) for comparison, including BP neural network (BPNN), SVM, KNN, and deep BP neural network (DBPN). In the traditional intelligent fault diagnosis methods, the manual feature extraction of a raw dataset is conducted firstly. Then, the extracted features are input into the intelligent diagnosis algorithm to complete the classification prediction [29]. Statistical features in the time domain and frequency domain are used in References [2,4,52]. Reference [4] extracted 14 features from raw data by using 14 feature extraction operators, including 10 time-domain features and 4 frequency-domain features; the detailed parameters are listed in Table 15 [4]. This paper is based on the 14 feature extraction operators to calculate the 14 statistical features of each bearing fault sample. Then, the extracted 14 features are input into SVM, KNN, and BPNN for a fault diagnosis. The experimental results of the precision rate and the recall rate of the six methods are listed in Table 16. Figure 20 shows that the six methods correspond to the F1-measure values (Percentage form).

Comparing Table 16 and Figure 20, it can be obviously seen that the accuracy of the proposed CNN-SVM algorithm is obviously better than other intelligent algorithms. In terms of accuracy, the precision rates of SVM, KNN, and BPNN after manual feature extraction are 94.95%, 91.15%, and 83.95%, respectively. The precision rate of DNN is 93.66% by using 4 hidden layers to train raw data. Only one hidden layer of BPNN has the lowest training accuracy and serious over-fitting. When the number of DNN layers exceeds 5 layers, the accuracy of DNN is difficult to increase because of the gradient disappearance, and the over-fitting of the model is gradually obvious. It can be seen that the recognition accuracy of the six algorithms for the normal state (condition 0) is high, but in terms of micro-fault identification (condition 1, condition 4, and condition 7), the CNN-SVM algorithm is significantly better than the other five algorithms. By comparing the results of the above six algorithms, it can be confirmed that the proposed CNN-SVM algorithm has a more superior performance. The proposed CNN-SVM method focuses on a rolling bearing intelligence fault diagnosis without any manual feature extraction or signal preprocessing, which is totally different from the traditional methods. 

The main parameters of the other methods are described as follows: (1) SVM: the penalty factor C = 10, kernel function is Gaussian radial basis function (RBF), and slack variable ξ=0.1; (2) KNN: using the Minkowski distance, the *k* value is 5 and the leaf node is 30; (3) BPNN: the architecture is 500–512–10, the Adam adaptive learning rate optimization algorithm is used, the activation function is Relu, the regularization coefficient λ = 0.001, the batch size is 64, and the iteration epoch is 300; and (4) DNN: the architecture is 500–512–256–128–128–10, which is decided by repeated trial experiences and guiding principles, the Adam optimization algorithm is used, the activation function is Relu, the regularization coefficient λ = 0.001, the batch size is 64, and the iteration epoch is 500. 

## 5. Conclusions 

As an emerging machine learning algorithm, deep learning is gradually applied in the field of intelligent fault diagnosis. In this paper, a novel method called the improved global average pooling CNN-SVM method is proposed for the intelligent diagnosis of incipient faults in rotating machinery. The proposed method improves the traditional CNN model structure, introduces the global average pooling technology to replace the fully connected network structure, and uses the SVM to replace the Softmax function in the test stage. Firstly, the multichannel raw fault data is directly input into the improved CNN-Softmax model to complete the training of CNN model by a back-propagation algorithm. Secondly, the improved CNN is used as feature extractors to extract representative features of the new fault raw data. Finally, the extracted sparse representative feature vectors are input into SVM that is used for fault classification. The proposed method can effectively reduce the CNN’s model parameter quantity and diagnosis time and can effectively improve the accuracy of fault diagnosis results.

The proposed method is applied to the diagnosis of rolling bearing multichannel experimental vibration signal data and compared with traditional intelligent diagnosis methods. The results confirm that the proposed CNN-SVM method need not any manual feature extraction and signal processing operations on the raw data during the whole diagnosis process. It can get rid of the dependence on manual feature extraction and can overcome the limitations of traditional methods relying on expert experience. The category and quantity of each fault type of is quantified and visualized by using a multi-class confusion matrix. The deep learning training skills are used to improve the algorithm performance including data enhancement, dropout, and batch normalization. The experiment result confirms the proposed method is more effective than other existing intelligence diagnosis methods including SVM, KNN, BPNN, DNN, and traditional CNN. The end-to-end model structure has a better operability and versatility. It is very interesting and innovative to use CNN-SVM and other deep learning methods to explore online real-time intelligence diagnosis and hardware implementation for new applications. The authors will continue to research this topic in the future. 

## Figures and Tables

**Table 1:** The commonly used activation functions in a convolutional neural network (CNN).

Function Name	Sigmoid	Tanh	Relu
expressions	$f(x)=\frac{1}{1+e^{-x}}$	$f(x)=\frac{1-e^{-2x}}{1+e^{-2x}}$	$f(x)=\{\begin{array}{c} x,\;x\geq 0\\ 0,\;x<0 \end{array}$
graphs	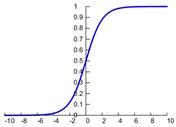	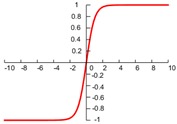	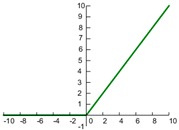

**Table 2:** The bearing size of the drive end and fan end.

Parameter Type	Inside Diameter	Outside Diameter	Thickness	Ball Diameter	Pitch Diameter
	Size: (inches)				
**Drive end bearing**	0.9843	2.0472	0.5906	0.3126	1.5370
**Fan end bearing**	0.6693	1.5748	0.4724	0.2656	1.1220

**Table 3:** The description of the rolling bearing multichannel fault dataset under 0–2 hp load condition.

Class Label	Fault Location	Fault Size (inches)	Fault Severity	Sample Length	Sample Number
0	Normal	None	None	500 × 2	200 × 3
1	Ball	Diameter: 0.007, Depth: 0.011	incipient fault (F^−^)	500 × 2	200 × 3
2	Ball	Diameter: 0.014, Depth: 0.011	moderate fault (F)	500 × 2	200 × 3
3	Ball	Diameter: 0.021, Depth: 0.011	significant fault (F^+^)	500 × 2	200 × 3
4	Inner Raceway	Diameter: 0.007, Depth: 0.011	incipient fault (F^−^)	500 × 2	200 × 3
5	Inner Raceway	Diameter: 0.014, Depth: 0.011	moderate fault (F)	500 × 2	200 × 3
6	Inner Raceway	Diameter: 0.021, Depth: 0.011	significant fault (F^+^)	500 × 2	200 × 3
7	Outer Raceway	Diameter: 0.007, Depth: 0.011	incipient fault (F^−^)	500 × 2	200 × 3
8	Outer Raceway	Diameter: 0.014, Depth: 0.011	moderate fault (F)	500 × 2	200 × 3
9	Outer Raceway	Diameter: 0.021, Depth: 0.011	significant fault (F^+^)	500 × 2	200 × 3

**Table 4:** The description of the dataset division.

Class Label	Fault Location	Sample Length	Sample Number	Training Dataset	Verification Dataset	Testing Dataset
0	Normal	500 × 2	200 × 3	112 × 3	28 × 3	60 × 3
1	Ball	500 × 2	200 × 3	112 × 3	28 × 3	60 × 3
2	Ball	500 × 2	200 × 3	112 × 3	28 × 3	60 × 3
3	Ball	500 × 2	200 × 3	112 × 3	28 × 3	60 × 3
4	Inner Raceway	500 × 2	200 × 3	112 × 3	28 × 3	60 × 3
5	Inner Raceway	500 × 2	200 × 3	112 × 3	28 × 3	60 × 3
6	Inner Raceway	500 × 2	200 × 3	112 × 3	28 × 3	60 × 3
7	Outer Raceway	500 × 2	200 × 3	112 × 3	28 × 3	60 × 3
8	Outer Raceway	500 × 2	200 × 3	112 × 3	28 × 3	60 × 3
9	Outer Raceway	500 × 2	200 × 3	112 × 3	28 × 3	60 × 3

**Table 5:** The descriptions of the hyper-parameters of the CNN reference model.

Layer Type	Hyper-Parameter Settings	Output Shape	Learnable Parameters
Input layer	(batch, 25 × 20, 2)	(batch, 25 × 20, 2)	0
Convolution layer 1	Filter = (3 × 3, 2, 64), strides = (1, 1), padding = “Same”	(batch, 25 × 20, 64)	1216
Activation layer 1	activation function	(batch, 25 × 20, 64)	0
Max-Pooling layer 1	Ksize = (1, 2 × 2, 1), strides = (2, 2), padding = “Same”	(batch, 12 × 10, 64)	0
Convolution layer 2	Filter = (3 × 3, 64, 32), strides = (1, 1), padding = “Same”	(batch, 12 × 10, 32)	18,464
Activation layer 2	activation function	(batch, 12 × 10, 32)	0
Max-Pooling layer 2	Ksize = (1, 2 × 2, 1), strides = (2, 2), padding = “Same”	(batch, 6 × 5, 32)	0
Flatten layer	flatten Max-Pooling layer 2 to 1-D shape	(batch, 960)	0
FC-Dense layer 1	128 hidden layer neuron nodes	(batch, 128)	123,008
FC-Activation layer 1	activation function	(batch, 128)	0
FC-Dense layer 2	10 hidden layer neuron nodes	(batch, 10)	1290
Softmax output layer	Softmax activation function	(batch, 10)	0

**Table 6:** The experiment results of the activation function.

Serial Number	Convolution Layer 1	Convolution Layer 2	Fully Connected Layer	Accuracy	Training Time (s)	Test Time (s)
1	relu	relu	relu	0.9768	117.104	0.1275
2	tanh	tanh	tanh	0.9814	120.652	0.1404
3	sigmoid	sigmoid	sigmoid	0.7309	121.302	0.1875
4	relu	relu	tanh	0.9897	117.392	0.1404
5	relu	relu	sigmoid	0.9692	124.728	0.1455
6	sigmoid	sigmoid	relu	0.3938	122.864	0.1572
7	sigmoid	sigmoid	tanh	0.3106	123.896	0.1704
8	tanh	tanh	relu	0.9683	123.924	0.1946
9	tanh	tanh	sigmoid	0.9692	123.416	0.1404

**Table 7:** The experiment result of different optimization algorithms.

Optimization Algorithm	Number of Iterations	Accuracy	Training Time (s)	Test Time (s)
SGD	100	0.9276	123.172	0.18936
Adagrad	100	0.9882	117.912	0.17145
AdaDelta	100	0.9871	124.252	0.18351
RMSProp	100	0.9872	114.092	0.16842
Adam	100	0.9899	114.876	0.16739

**Table 8:** The experiment results of the different mini-batch numbers.

Mini-Batch Number	Accuracy	Average Training Time (s)
1	0.9585	4286.88
8	0.9799	601.056
16	0.9872	324.976
32	0.9883	185.288
64	0.9899	114.876
128	0.9828	87.6292
256	0.9640	69.3592
512	0.8729	57.9404

**Table 9:** The hyper-parameters of the CNN methods.

CNN	CNN Model Hyper-Parameters
L = 1	Conv2d (3 × 3, 64) + Pooling (2 × 2) + Full Connect (128/10) + Softmax
	Conv2d (3 × 3, 64) + Pooling (2 × 2) + Dropout (0.5) + Full Connect (128/10) + Softmax
L = 2	Conv2d (3 × 3, 64) + Pooling (2 × 2) + Conv2d (3 × 3, 32) + Pooling (2 × 2) + Full Connect (128/10) + Softmax
	Data augmentation + Conv2d (3 × 3, 64) + Pooling (2 × 2) + Dropout (0.3) + Conv2d (3 × 3, 32) + Pooling (2 × 2) + Dropout (0.2) + Full Connect (128/10) + Softmax
L = 3	Data augmentation + Conv2d (3 × 3, 64) + Pooling (2 × 2) + Conv2d (3 × 3, 32) + Pooling (2 × 2) + Conv2d (3 × 3, 32) + Pooling (2 × 2) + Full Connect (128/10) + Softmax
	Data augmentation + Conv2d (3 × 3, 64) + Pooling (2 × 2) + Dropout (0.3) + Conv2d (3 × 3, 32) + Pooling (2 × 2) + Dropout (0.2) + Conv2d (3 × 3, 32) + Pooling (2 × 2) + Dropout (0.2) + Full Connect (128/10) + Softmax

**Table 10:** The accuracy of different CNN models for fault identification.

Model Structure	L1	L2	L3
	Test Accuracy		
**No training skills**	96.04%	98.31%	98.76%
**Data augmentation + Dropout**	97.92%	98.99%	98.93%

**Table 11:** The details of the proposed CNN-SVM model hyper-parameters in the experiments.

CNN-Softmax Training Stage Model Structure			
Layer Type	Parameter Settings	Output Shape	Learnable Parameters
Input layer	(batch, 25 × 20, 2)	(batch, 25 × 20, 2)	0
Convolution layer 1	Filter = (3 × 3, 2, 64), strides = (1, 1), padding = “Same”	(batch, 25 × 20, 64)	1216
Activation layer 1	ReLU activation function	(batch, 25 × 20, 64)	0
Max-Pooling layer 1	ksize = (1, 2 × 2, 1), strides = (2, 2), padding = “Same”	(batch, 12 × 10, 64)	0
Dropout layer 1	Dropout (0.3)	(batch, 12 × 10, 64)	0
Convolution layer 2	filter = (3 × 3, 64, 32), strides = (1, 1), padding = “Same”	(batch, 12 × 10, 32)	18,464
Activation layer 2	ReLU activation function	(batch, 12 × 10, 32)	0
Max-Pooling layer 2	ksize = (1, 2 × 2, 1), strides = (2, 2), padding = “Same”	(batch, 6 × 5, 32)	0
Dropout layer 2	Dropout (0.2)	(batch, 6 × 5, 32)	0
Convolution layer 3	filter = (1 × 1, 32, 10), strides = (1, 1), padding = “Same”	(batch, 6 × 5, 10)	330
Global average pooling layer	ksize = (1, 5 × 4, 1), strides = (5, 4), padding = “Same”	(batch, 10)	0
CNN feature output layer	Save CNN model parameters	(batch, 10)	0
Softmax output layer	Softmax activation function	(batch, 10)	0
CNN-SVM test stage model structure			
Input layer	(batch, 25 × 20, 2)	(batch, 25 × 20, 2)	0
CNN feature output layer	raw data input into trained CNN extraction features	(batch, 10)	0
SVM classification layer	Nonlinear SVM classifier	(batch, 10)	110
Final output layer	Fault diagnosis result	(batch, 10)	0

**Table 12:** A comparison of the trainable parameter quantities between the traditional CNN model and the proposed CNN-SVM model.

Layer Type	Traditional Fully Connected CNN Model	Improved CNN-SVM
Convolution layer 1	1216	1216
Convolution layer 2	18,464	18,464
Convolution layer 3	None	330
FC-Dense layer 1	123,008	None
FC-Dense layer 2	1290	None
SVM classification layer	None	110
Total parameter amount	143,978	20,120

**Table 13:** The fault diagnosis results of the proposed CNN-SVM model.

Model Name	Test Accuracy	Training Time (s)	Testing Time (s)
Improved CNN+Softmax	99.12%	73.8293	0.08967
Improved CNN+SVM	99.94%	74.3205	0.08318

**Table 14:** The evaluation results of the proposed CNN-SVM model.

Health Condition	Precision Rate	Recall Rate	F1-Measure	Sample Amount
Condition 0	100%	100%	100%	180
Condition 1	100%	100%	100%	180
Condition 2	100%	99.44%	99.72%	180
Condition 3	100%	100%	100%	180
Condition 4	100%	100%	100%	180
Condition 5	100%	100%	100%	180
Condition 6	100%	100%	100%	180
Condition 7	100%	100%	100%	180
Condition 8	99.45%	100%	99.72%	180
Condition 9	100%	100%	100%	180
Average/total	99.94%	99.94%	99.94%	1800

**Table 15:** The statistical feature extractions in the time-domain and frequency-domain.

Domain	Feature Parameters	
Time-domain	Absolute mean: $\frac{1}{n}\sum_{i=1}^{n}\left|x_{i}\right|$	Crest factor: $\max\left(\left|x_{i}\right|\right)/\sqrt{\frac{1}{n}\sum_{i=1}^{n}x_{i}{}^{2}}$
	Variance: $\frac{1}{n}\sum_{i=1}^{n}{\left(x_{i}-\bar{x}\right)}^{2}$	Root mean square: $\sqrt{\frac{1}{n}\sum_{i=1}^{n}x_{i}{}^{2}}$
	Crest: $\max\left(\left|x_{i}\right|\right)$	Pulse factor: $\max\left(\left|x_{i}\right|\right)/\left(\frac{1}{n}\sum_{i=1}^{n}\left|x_{i}\right|\right)$
	Clearance factor: $\max\left(\left|x_{i}\right|\right)/{\left(\frac{1}{n}\sum_{i=1}^{n}\sqrt{x_{i}{}^{2}}\right)}^{2}$	Skewness: $\frac{1}{n}\sum_{i=1}^{n}x_{i}{}^{3}$
	Kurtosis: $\frac{1}{n}\sum_{i=1}^{n}x_{i}{}^{4}$	Shape factor: $\sqrt{\frac{1}{n}\sum_{i=1}^{n}x_{i}{}^{2}}/\left(\frac{1}{n}\sum_{i=1}^{n}\left|x_{i}\right|\right)$
Frequency-domain	Crest: $\max\left(\left|X_{i}\right|\right)$	Mean energy: $\frac{1}{n}\sum_{i=1}^{n}X_{i}{}^{}$
	Kurtosis: $\frac{1}{n}\sum_{i=1}^{n}X_{i}{}^{4}$	Variance: $\frac{1}{n}\sum_{i=1}^{n}{\left(X_{i}-\bar{X}\right)}^{2}$

**Table 16:** The precision rate and recall rate of different methods.

	Methods											
Bearing Condition	CNN-SVM		SVM		KNN		BPNN		DNN		CNN	
	*P* (%)	*R* (%)	*P* (%)	*R* (%)	*P* (%)	*R* (%)	*P* (%)	*R* (%)	*P* (%)	*R* (%)	*P* (%)	*R* (%)
Condition 0	100	100	100	100	100	97.78	91.24	98.33	99.45	100	100	100
Condition 1	100	100	89.67	91.67	98.29	95.56	90.66	91.67	76.50	99.44	95.72	99.44
Condition 2	100	99.44	90.34	88.33	87.28	83.89	72.40	77.22	69.77	100	94.74	100
Condition 3	100	100	86.75	80.01	91.47	65.56	75.01	45.01	93.33	54.44	100	90.56
Condition 4	100	100	95.24	100	82.24	69.44	99.42	95.56	100	98.89	100	100
Condition 5	100	100	98.85	95.56	84.11	100	100	100	100	89.44	100	100
Condition 6	100	100	94.15	98.33	71.43	100	91.84	100	98.08	85.00	100	100
Condition 7	100	100	99.45	100	100	100	64.98	92.78	100	93.89	100	100
Condition 8	99.45	100	95.58	96.11	100	92.22	76.00	84.44	99.45	100	99.44	99.44
Condition 9	100	100	99.45	100	96.72	98.33	77.97	51.11	100	98.33	100	100
Average value	99.94	99.94	94.95	95.01	91.15	90.28	83.95	83.61	93.66	91.94	98.99	98.94
